# Targeting miR-5088-5p attenuates radioresistance by suppressing Slug

**DOI:** 10.1016/j.ncrna.2022.12.005

**Published:** 2023-01-02

**Authors:** Hyun Jeong Seok, Jae Yeon Choi, Joo Mi Yi, In Hwa Bae

**Affiliations:** a Division of Radiation Biomedical Research, Korea Institute of Radiological & Medical Sciences, Seoul, Republic of Korea; b Department of Microbiology and Immunology, College of Medicine, Inje University, Busan, Republic of Korea

**Keywords:** miR-5088-5p inhibitor, Radioresistance, Resistance, Slug, Promoter methylation, IR, ionizing radiation, miRNA, microRNA, DBC2, deleted in breast cancer 2, DNMT, DNA methyl transferases, MSP, methylation-specific PCR, VEGF, vascular endothelial growth factor, Ang, angiopoietin, CSC, cancer stem-like cell, EMT, epithelial-mesenchymal transition, H&E, hematoxylin and eosin, MTT, methylthiazole tetrazolium, siRNA, small-interfering RNA

## Abstract

Radiotherapy is widely used for cancer treatment, but paradoxically, it has been reported that surviving cancer cells can acquire resistance, leading to recurrence or metastasis. Efforts to reduce radioresistance are required to increase the effectiveness of radiotherapy. miRNAs are advantageous as therapeutic agents because it can simultaneously inhibit the expression of several target mRNAs. Therefore, this study discovered miRNA that regulated radioresistance and elucidated its signaling mechanism. Our previous study confirmed that miR-5088-5p was associated with malignancy and metastasis in breast cancer. As a study to clarify the relationship between radiation and miR-5088-5p identified as onco-miRNA, it was confirmed that radiation induced hypomethylation of the promoter of miR-5088-5p and its expression increased. On the other hand, miR-5088-5p inhibitors were confirmed to reduce radiation-induced epithelial-mesenchymal transition, stemness, and metastasis by reducing Slug. Therefore, this study showed the potential of miR-5088-5p inhibitors as therapeutic agents to suppress radioresistance.

## 1 Introduction

Radiotherapy is commonly used for treatment of about 50% of all cancer patients. In particular, radiotherapy is efficacious at the early stages of development of various tumor types, including skin, prostate, lung, cervical, lymphoma, and head and neck [1]. However, ionizing radiation (IR) is well established carcinogen [2] with occurrence of secondary malignant cancers documented in patients with primary carcinoma exposed to radiotherapy [[3], [4], [5]]. Increased risk of radiation-induced malignancy has additionally been reported in breast, prostate, gynecological tumor types, lymphoma, and pediatric malignancies [[6], [7], [8], [9], [10], [11]]. To enhance the efficiency of radiotherapy, reduction of radiation-induced malignancy remains an urgent clinical requirement [12]. The current study explored the utility of microRNAs (miRNA) as therapeutic targets to inhibit the side-effects of radiation based on identification of radiation-associated target miRNAs and their underlying mechanisms.

MiRNA is a type of non-coding RNA composed of 18–24 nucleotides that regulates post-transcriptional expression of target genes. MiRNAs are involved in numerous biological pathways and cellular processes, including cell proliferation, apoptosis, development, and cell signaling [13]. Depending on the tumor type, miRNAs can function as either onco-miRNAs or tumor suppressor genes (anti-onco-miRNAs) [14,15]. In view of previous findings that deregulation or dysfunction of miRNAs contributes to cancer development, their efficacy as a cancer biomarker has been actively researched [13,[16], [17], [18]]. For example, miR-224 [19], miR-130b [20], and miR-144-3p [21] are known to increase metastasis in hepatocellular carcinoma, non-small cell lung cancer, and renal cell carcinoma, respectively. These molecules are released from cells and circulate in the blood, presenting an efficient tool for diagnosis of multiple diseases including cancer [13,22].

Several studies have reported changes in IR-induced miRNA expression profiling [[23], [24], [25], [26], [27], [28], [29], [30], [31], [32], [33]]. Based on previous papers, a list of miRNAs that are induced or decreased in response to IR was compiled (Table 1) [[23], [24], [25], [26], [27], [28], [29], [30], [31], [32], [33]]. For example, radiation-upregulated miR-21 [23] and down-regulated miR-143 [24] are associated with radiation-induced carcinogenesis, suggesting critical roles in radiation-mediated malignancy. Also, an inhibitor of miRNA-21 [26], reported to be induced by radiation, has been reported to increase radiation-induced cell death by increasing radio-sensitivity through inhibiting pAKT in malignant glioma cell lines [34]. As an advantage of miRNA, it is known as an efficient tool for diagnosing various diseases including cancer by being released from cells and circulating in the blood [13,22]. Therefore, these studies based on radiation and miRNAs suggest a possibility as diagnostic and therapeutic targets that increase the efficiency of radiotherapy.

**Table 1:** List of miRNAs that are differentially regulated in response to IR.

IR-induced miRNAs	IR-decreased miRNAs
miR-17-3p [21]	miR-15 a/b [22]
miR-21 [26]	miR-16 [23]
miR-22 [22]	miR-143 [27]
miR-29c [23]	miR-195 [22]
miR-34a [23]	miR-424 [22]
miR-99 [29]	miR-497 [28]
miR-100 [29]	
miR-143 [21]	
miR-145 [21]	
miR-193a-3p [24]	
miR-193b [23]	
miR-365 [23]	
miR-494 [30]	

Experiments by our group confirmed that miR-5088-5p, a miRNA identified in a previous study, promotes malignancy by inhibiting the expression of Deleted in Breast Cancer 2 (DBC2) in breast cancer [25]. The current study focused on the relationship between onco-miR-5088-5p and IR-induced malignancy. It was found that IR increases the expression of miR-5088-5p through promoter hypomethylation and decreases the expression of its target DBC2, eventually promoting tumor progression. Therefore, IR-induced tumorigenicity was effectively inhibited by treatment with a miR-5088-5p inhibitor. This supports the potential of miR-5088-5p inhibitor as a combination therapeutic agent to improve radiotherapy efficiency.

## 2 Materials and methods

### 2.1 Cell culture

Highly metastatic MDA-MB-231 cells provided by S.J. Lee (FNCT Biotech, Korea) laboratory. MDA-MB-231 and H460 cells obtained from the Korea Cell Line Bank (KCLB, Korea). H460 and MDA-MB-231 cells were cultured in RPMI 1640 media (Corning, NY) and DMEM (Corning, NY) with 10% FBS (Corning, NY) and 1% penicillin-streptomycin antibiotics (PAA Laboratories GmbH, Austria), respectively. All cells were cultured in a 5% CO_2_ incubator at 37 °C. All cell lines were routinely tested for mycoplasma using the e-Myco™ Mycoplasma PCR Detection Kit (iNtRON's, Korea) prior to use in experiments.

### 2.2 RNA extraction and qRT-PCR

Plasma was isolated from normal and breast cancer patients and metastasis mouse model by 13,000 rpm, 15 min centrifugation. Total RNA was extracted with TRIzol reagent (Molecular Research Center Inc., OH, USA) from plasma obtained from patients and mice. The miRNA was synthesized using the Mir-X miRNA First-Stand cDNA Synthesis Kit (Takara, Japan) and the mRNA using the Tetro cDNA Synthesis Kit (BIOLINE, London, UK). Real time PCR was quantified with U6 (Takara, Japan) and GAPDH. All data were analyzed by the 2^-△△*Cr*^ method. The sequences of the primers are listed in Table 2.

**Table 2:** Primer sequences used in this study.

Primers for qRT-PCR	Sequences (5′-3′)	
**primary-miR-5088-5p**	Forward	CCTCTGCATGTTTGCTGCCA
	Reverse	TGAGGGCCCAGGAAGAAGGGA
**precursor-miR-5088-5p**	Forward	CAGGGCTCAGGGATTGGATGGAGG
	Reverse	TGAGGGCCCAGGAAGAAGGGA
**mature-miR-5088-5p**	Forward	CAGGGCTCAGGGATTGGATGGAGG
**MMP-2**	Forward	CATCAAGGGCATTCAGGAGC
	Reverse	AGAACACAGCCTTCTCCTCC
**MMP-9**	Forward	AGGACGACGTGAATGGCATG
	Reverse	ATCGTCCACCGGACTCAAAG
**VEGF**	Forward	GACAGACAGACAGACACCGCC
	Reverse	GAACAGCCCA GAAGTTGGACG
**Ang2**	Forward	GCAAGTGCTGGAGAACATCA
	Reverse	CACAGCCGTCTGGTTCTGTA
**Oct4**	Forward	GAGCAAAACCCGG AGGAGT
	Reverse	TTCTCTTTCGGGCCTGCAC
**Nanog**	Forward	GCTTGCCTTGCT TTGAAGCA
	Reverse	GTTCTTGCATCTGCTGGAGG
**Sox2**	Forward	ATGCACCGCTACGACGTGA
	Reverse	CTTTTGCACCCCTCCCATT
**CD133**	Forward	GCCACCGCTCTAGATACTGC
	Reverse	GCTTTTCCTATGCCAAACCA
**CD44**	Forward	AAGGTGGAGCAAACACAACC
	Reverse	ACTGCAATGCAAACTGCAAG
**DBC2**	Forward	CAGAGCAGTAGACAGTGACC
	Reverse	TGTAGTTGGTGCAGATGTGG
**GAPDH**	Forward	CATCTCTGCCCCCTCTGCTGA
	Reverse	GGATGACCTTGCCCACAGCCT
**Slug**	Forward	ACAGCGAACTGGACACACAT
	Reverse	TCACTCGCCCCAAAGATGAG
**Snail**	Forward	GTTTACCTTCCAGCAGCCCT
	Reverse	GAGCCTTTCCCACTGTCCTC
**Zeb1**	Forward	GCCAATAAGCAAACGATTCTG
	Reverse	TTTGGCTGGATCACTTTCAAG
**Vimentin**	Forward	GCTTGGAAACATCCACATCG
	Reverse	GAGAGGAAGCCGAAAACACC
**N-cadherin**	Forward	CCTGGAACGCAGTGTACAGA
	Reverse	TGGTTTGACCACGGTGACTA
Primers for overexpression vector	**Sequences (5′-3′)**	
**Slug overexpression**	Forward	CGCGGATCCATGCCGCGCTCCTTCCTG
	Reverse	CGGAATTCTCAGTGTGCTACACAGCAGCCA
Primers for pyrosequencing analysis	**Sequences (5′-3′)**	
**Methy-miR-5088-5p**	Forward	ATTTTTGTTTTAGGAGATGTTGAAG
	Reverse	CCTAACCACCCTAAAAACTCA
Primers for MSP and qMSP	**Sequences (5′-3′)**	
**Unmethylation miR-5088-5p**	Forward	GTTTTAGGAGATGTTGAAGGATGA
	Reverse	CATACAAAAAATACCAATACCCAAA
**Methylation miR-5088-5p**	Forward	TTTGTTTTAGGAGATGTTGAAGGAC
	Reverse	ATACAAAAAATACCGATACCCGAA

### 2.3 Immunoblot analysis

Cells were lysed with RIPA buffer containing protease and phosphatase inhibitor cocktails (Roche, Indianapolis, USA). The extracted protein concentration was measured using Bradford assay (Bio-Rad Laboratories Inc, CA, USA). Proteins were separated using SDS-PAGE and then transferred to PVDF (Millipore Corporation, MA, USA). The membrane was blocked with 5% BSA (bovine serum albumin) in Tris-buffered saline-Tween 20 (TBST) (10 mM TrisHCl, pH 8.0, 150 mM NaCl and 0.05% Tween 20) and the primary antibody was treated. The secondary antibody was treated by dilution in 5% skim milk. After washing with TBST, it was detected with ECL solution (Thermo Scientific, Pierce, USA) using AmershamTM Imager 600 system equipment (GE Healthcare Bio-Sciences, PA, USA).

Antibodies against Slug and β-actin were obtained from Santa Cruz Biotechnology (CA, USA). Zeb1 were purchased from Sigma (Sigma-Aldrich Company, MO, USA). Twist and N-cadherin were purchased from Abcam Inc. (Cambridge, MA, USA). Snail, Vimentin, DNMT1, DNMT3a, and DNMT3b were purchased from Cell Signaling technology (Beverly, MA, USA).

### 2.4 Irradiation

Ionizing radiation (IR) was performed H460 and MDA-MB-231 cells in 60 mm dishes as 5Gy and cells were harvested after 48 h. We irradiated with a 3.81 Gy/min dose rate using a 137Cs γ-rays source (Atomic Energy of Canada, Ltd, Canada). The mouse model is irradiated at 2 Gy/min dose rate using X-RAD 320 irradiator (PXi, North Branford, CT) equipment.

### 2.5 Plasmid DNA, RNA oligoribonucleotides, and transfection

To make Slug-overexpressing vector, the Slug gene was inserted to the pCMV-taq2 vector. The sequences of primers used for overexpression are listed in Table 2. The inhibitor of miR-5088-5p was a 2′-O-methyl-modified oligoribonucleotide single strand with the sequence as 5′-CCUCCAUCCAA UCCCUGAGCCCU-3′ and was synthesized by IDT (Integrated DNA Technologies, Coralville, IA, USA). All siRNAs (Slug, Zeb1, and Snail) were purchased from Santa Cruz Biotechnology (Santa Cruz, CA, USA). siRNAs (20 mM), miRNAs (10 mM), and plasmids (4 μg) were introduced into cells using G-fectin (Genolution, Seoul, Korea) or Lipofectamine 2000 reagent (Thermo Fisher Scientific, Invitrogen, USA) according to the manufacturer's instructions, respectively.

### 2.6 Wound healing assay

Cells were seeded on 6-well culture plates and a plastic tip was scraped to mimic wound damage. Cells were washed with PBS to remove cell debris and moved for 16–24 h. The recovery of wound damage was quantified by counting cells after observation with a light microscope (CKX53, OLYMPUS, Tokyo, Japan) [26].

### 2.7 Transwell invasion assay

After coating matrigel (BD Biosciences, CA, USA) in a transwell chamber (8 μm pore) (Corning, NY), transfected cells were placed in the upper transwell chamber with opti-MEM (Gibco, USA) medium. After culture media was added to the lower chamber and cultured for 16 h, the invaded cells in the lower transwell chamber were fixed and stained with Hemacolor solution (Merck, MA, USA). Stained cells were counted under a light microscope (CKX53, OLYMPUS, Tokyo, Japan).

### 2.8 Tube formation assay

The supernatant of MDA-MB-231 cells transfected with negative control (NC) or miR-5088-5p inhibitor using G-fectin (Genolution, Seoul, Korea) and/or IR 5Gy combination was harvested after 72 h. After coating a 96-well plate with matrigel, 1 × 10^4^ cells/well of HUVECs and supernatant obtained from MDA-MB-231 cells were simultaneously added to the plate. After 16 h, tube forming ability was observed under a microscope (Miotic AE31 series, Motic, Hong Kong).

### 2.9 Sphere formation assay

After transfection of MDA-MB-231 cells (1 × 10^5^/dish) with negative control (NC) or miR-5088-5p inhibitor, cells seeded in Dulbecco's Modified Eagle Medium-F12 (Gibco, USA) containing B27 (1:50) (Gibco, USA) and grown for 7–10 days. Spheres (>20 μm in diameter) were counted in an inverted microscope (Miotic AE31 series, Motic, Hong Kong).

### 2.10 In vivo promoter methylation analysis

In the mouse xenograft model, BALB/c nude mice (female, 6 weeks old) from Envigo were injected subcutaneously with H460 (1 × 10^7^ cells). After 14 days, 2.5Gy of radiation was fractionated to the mouse's chest every day for 3 days. After 56 days, the mouse was sacrificed, and tumors generated from the subcutaneous tissue were isolated and ground to extract genomic DNA and mRNA. Methylation of miR-5088-5p promoter was confirmed using genomic DNA by phenol-chloroform extraction method, and the level of miR-5088-5p was confirmed using mRNA by TRIzol method. This experiment was reviewed and approved by the Institutional Animal Care and Use Committee (IACUC) of Korea Institute of Radiological & Medical Science.

### 2.11 Pyrosequencing analysis

To confirm the methylation of miR-5088-5p at 10 sites on the CpG islands of the miR-5088-5p promoter, pyrosequencing analysis was performed. PCR was performed with taq polymerase (TaKaRa, Kyoto, Japan) using the primers of miR-5088-5p. The sequences of the methy-miR-5088-5p primers used for pyrosequencing analysis are listed in Table 2 [25]. Pyrosequencing was conducted on a PyroMark ID system (Qiagen, Hilden, Germany) using streptavidin Sepharose HP beads (Amersham Biosciences, Piscataway, NJ) and a Pyro Gold Kit (Qiagen, Hilden, Germany) according to the manufacturer's instructions.

### 2.12 Methylation specific PCR (MSP) and quantitative methylation-specific PCR (qMSP) analysis

After genomic DNA was extracted according to a standard phenol-chloroform extraction method, bisulfite modification of genomic DNA was performed using an EZ DNA methylation kit (Zymo Research, USA). The methylation analysis of the miR-5088-5p promoters was performed using MSP primer pairs covering the putative transcriptional start site in the 5′ CpG islands with 1 μℓ of bisulfite-treated DNA as template and JumpStart Red Taq DNA Polymerase (Sigma-Aldrich Company, MO, USA) for amplification as previously described [27]. DKO (DNMT1(−/−), DNMT3B (−/−) double knockout in HCT116 cells) as unmethylation control, IVD (in vitro methylated DNA) as methylation control, and ddH2O as PCR negative control were used. Primers of the miR-5088-5p promoter region across the upstream from −225 to 34 from mature form sites (Supplementary Fig. S3A). The sequences of the unmethylation and methylation miR-5088-5p promoter primers used for MSP and qMSP are listed in Table 2. MSP amplification was performed on bisulfite treated samples and normalized using the Alu element. Real-time PCR was performed by a CFX96TM real-time system (Bio-Rad, Hercules, CA, USA). Alu primer sequence information is previously described [28].

### 2.13 In vivo metastasis assay

Human lung cancer cells, H460 cells were transfected with negative control (NC) and anti-miR-5088-5p. 2 × 10^6^ cells were injected into tail vein of a six-week-old mouse. After 14 days, 2.5Gy of radiation was fractionated to the mouse's chest every day for 3 days. After 56 days, the mouse was sacrificed, and blood and lungs were collected. The lungs were fixed with 4% paraformaldehyde, metastatic nodules were counted, and a block was made to perform hematoxylin and eosin (H&E) staining (shandonTM, Thermo Scientific, Pierce, USA). This experiment was reviewed and approved by the Institutional Animal Care and Use Committee (IACUC) of Korea Institute of Radiological & Medical Science.

### 2.14 Colony formation assay

Cells (5 × 10^2^) were seeded in 60 mm dishes cultured for 3 weeks. When visible colonies appeared, the cells were washed twice with PBS, fixed in methanol for 15 min, stained with crystal violet for 20 min, washed with water, and air dried. The number of dishes repeated 3 times was counted. The number of visible colonies is the rate of colony formation = (number of colonies/number of seeding cells) × 100%.

### 2.15 MTT assay

H460 and MDA-MB-231 cells (5 × 10^3^ cells per well) were seeding on 96-well plates in triplicate wells. The next day, the cells were treated with 50 μM of cisplatin (Sigma-Aldrich Company, MO, USA) or 5Gy radiation. MTT reagent (3-(4,5-dimethylthiazol-2-yl)-2,5-diphenyltetrazolium bromide, Sigma-Aldrich Company, MO, USA) was added at 24 h, 48 h, and 72 h, and proliferation was measured at 450 nm after 4 h.

### 2.16 Clinical specimens

Biospecimens and data used in this study were provided by the Radiation Biobank (KRB) of Korea Cancer Center Hospital affiliated with the Korea Institute of Radiological and Medical Sciences (KIRAMS) in Republic of Korea. Breast cancer patient plasma was provided in samples from normal (n = 26) and breast cancer patients with (n = 15) and without (n = 21) radiotherapy, respectively (KRB-2016-I005). Lung cancer patient plasma was provided in samples from cancer patients with (n = 26) and without (n = 26) radiotherapy (KRB-2021-I002). All samples used in this experiment have Institutional Review Board (IRB) approval (K-1907-002-002, KIRAMS 2021-04-002-001) in KIRAMS.

### 2.17 Statistical analysis

All experiments were performed in triplicate. Data are presented as mean standard deviations and, where appropriate, Student's t tests were analyzed using GraphPad Prism software. P values of P < 0.05 were considered by Student *t*-test to be statistically significant.

## 3 Results

### 3.1 IR upregulates biogenesis of miR-5088-5p through inhibiting methylation

**Fig. 1:**
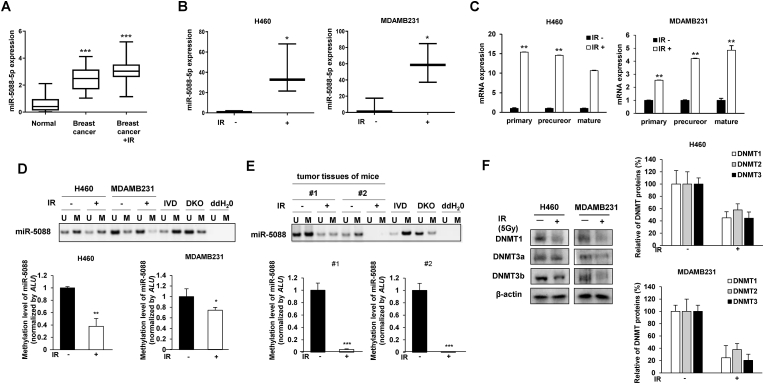
IR increases the biogenesis of miR-5088-5p via suppression of its methylation. (A) qRT-PCR analysis of miR-5088-5p expression in plasma of normal (n = 26) and breast cancer patients with (n = 15) and without (n = 21) radiotherapy. (B) Six-week-old mice were subcutaneously injected with H460 and MDA-MB-231 cells (1 × 10^7^ cells/mouse). At 2 weeks after injection, mice were irradiated with 2.5Gy/day for 3days. After 8weeks, mice were sacrificed, tumor tissue isolated and miR-5088-5p expression was confirmed via qRT-PCR (n = 3 out of 5). (C) After irradiation (5Gy) of H460 and MDA-MB-231 cells, levels of the primary, precursor, and mature forms of miR-5088-5p were determined by qRT-PCR and normalized to that of *U6*. (D) MSP and qMSP assays for methylation of CpG within the miR-5088-5p promoter region in H460 and MDA-MB-231 cells exposed to IR (5Gy). MSP analysis revealed unmethylated (U) and methylated (M) CpG sites and methylation of the *Alu* element was quantified via qMSP analysis. IVD, in vitro methylated control; DKO, DNMT1 (−/−) DNMT3b (−/−) double knockout (DKO) in HCT116 cells; ddHO, water control without DNA. (E) Confirmation of miR-5088-5p promoter methylation with MSP and qMSP assays using H460 subcutaneous tumor tissues of mice as for B (n = 5). (F) Immunoblot analysis of DNA methylation-related enzymes (DNMT1, DNMT3a, and DNMT3b) after IR (5 Gy) exposure (left) and quantification of bands by Image J program (right). β-actin was used as a loading control. All experiments were performed three times. Data are presented as the mean ± S.D. *P < 0.05, **P < 0.001, and ***P < 0.001 compared to control. Student's *t*-test.

Radiotherapy is one of the most common treatment options for cancer. A previous study by our group showed that miR-5088-5p promotes tumorigenic properties in breast cancer [25]. Here, we further examined the expression patterns and mechanisms of action of miR-5088-5p in tumor malignancy during radiotherapy. First, we compared the expression patterns of miR-5088-5p in plasma from breast and lung cancer patients with and without radiotherapy. As a result, miR-5088-5p was upregulated in plasma of breast or lung cancer patients treated with radiation compared to the non-radiated groups (Fig. 1A) and (Supplementary Fig. S1). In addition, expression of miR-5088-5p in the tumor tissues of irradiated mice after injection of lung and breast cancer cells, H460 or MDA-MB-231, was higher than that of the control group (Fig. 1B). In our previous study, our group identified the mechanism of DBC2 (Deleted in Breast Cancer 2) as a target of miR-5088-5p [25]. Therefore, to confirm the correlation between the two factors in this study, the expression of DBC2 by IR was confirmed. Expression of DBC2 was dramatically decreased in the tumor tissue exposed to radiation in the xenograft mouse model (Supplementary Fig. S2). This suggests that miR-5088-5p is up-regulated while DBC2 is down-regulated under IR conditions. According to these results, a negative correlation between miR-5088-5p and DBC2 was confirmed.

At the cellular level, the effect of radiation on biogenesis of miR-5088-5p was confirmed in H460 and MDA-MB-231 cells, respectively. Ionizing radiation (IR, 5Gy) enhanced biogenesis of miR-5088-5p by increasing the primary and precursor as well as mature forms of miR-5088-5p (Fig. 1C). Radiation is known to induce various epigenetic mutations in cancer [29]. Accordingly, epigenetic alterations of the miR-5088-5p promoter were further examined using MSP (methylation-specific PCR) and qMSP (quantitative methylation-specific PCR) to identify the underlying cause of upregulation. Reduction in methylation of the miR-5088-5p promoter via radiation was detected at the cellular level (Fig. 1D). The methylation of the miR-5088-5p promoter, including location of CpG islands, was additionally confirmed using pyrosequencing (Supplementary Fig. S3A). As a result, it was confirmed that IR reduced the methylation of 10 CpG islands in the miR-5088-5p promoter (Supplementary Fig. S3B). On the other hand, miR-5088-5p promoter methylation was decreased (Supplementary Fig. S4A) and expression of miR-5088-5p was increased (Supplementary Fig. S4B) in metastatic MDA-MB-231 relative to in parental MDA-MB-231 cells. Metastatic MDA-MB-231 (MDA-MB-231 LM2) cells constitute MDA-MB-231 cells that have metastasized to the lungs and are organ-specific metastatic clones with strong migration and invasive properties [30]. These results support our hypothesis that miR-5088-5p expression is upregulated via suppression of its promoter methylation, which, in turn, promotes tumor malignancy. For confirmation of our findings in an animal model, methylation of the miR-5088-5p promoter was examined in tumor tissues of mice with and without radiation treatment after subcutaneous injection of H460 cells. Decreased miR-5088-5p promoter methylation was confirmed in tumor tissues of radiation-treated relative to the non-irradiated mice (Fig. 1E).

In addition, IR induced a decrease in the expression of DNA methyl transferases (DNMT), such as DNMT1, DNMT3a, and DNMT3b, involved in methylation of H460 and MDA-MB-231 cells (Fig. 1F). Taken together, the data clearly indicate that IR decreased methylation of CpG islands in the miR-5088-5p promoter region via reducing DNMT, eventually inducing an increase in miR-5088-5p biosynthesis.

### 3.2 The miR-5088-5p inhibitor suppress IR-induced tumorigenic properties

**Fig. 2:**
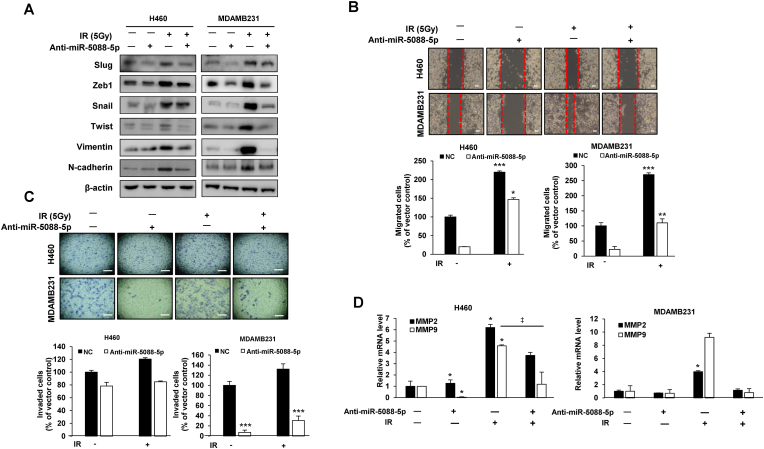
The miR-5088-5p inhibitor reduces IR-induced EMT, migratory ability, and invasiveness. (A–C) After IR (5Gy) exposure, H460 and MDA-MB-231 cells were transfected with or without miR-5088-5p inhibitor (anti-miR-5088-5p). (A) Immunoblot analysis of mesenchymal marker proteins (Slug, Zeb1, Snail, Twist, Slug, Vimentin, and N-cadherin) using β-actin as a loading control. (B) Wound healing assay of the migratory ability of cells (scale bar, 100 μm). (C) Matrigel-transwell assay of invasion ability (scale bar, 100 μm). (D) qRT-PCR analysis of invasion-related enzymes (MMP-2 and MMP-9). All assays were performed three times. The data are presented as the mean ± S.D. *P* values are *P < 0.05, **P < 0.01, and ***P < 0.001 compared to control; ‡*P* < 0.05 compared to IR group. Student's *t*-test.

We further examined whether miR-5088-5p is involved in the mechanism of IR-induced malignant transformation with the aid of an miR-5088-5p inhibitor. The malignant phenotype was confirmed after IR (5Gy) exposure of H460 and MDA-MB-231 cells transfected with miR-5088-5p inhibitor (anti-miR-5088-5p) or negative control. The miR-5088-5p inhibitor reduced IR-induced mesenchymal markers, such as Slug, Zeb1, Snail, Twist, Vimentin, and N-cadherin (Fig. 2A), cell mobility (Fig. 2B). MMP2 (matrix metalloproteinase-2, gelatinase A) and MMP9 (matrix metallopeptidase 9, gelatinase B) are enzymes that degrade type IV collagen, the most abundant component of the basement membrane, and are known to play an important role in the invasion process via EMT [[31], [32], [33]]. Therefore, we checked the expression of MMP2 and MMP9 as markers to confirm IR-induced invasion. As a results, the expression of MMP 2 and MMP 9 mRNA, which were increased by IR, were decreased by miR-5088-5p inhibitor (Fig. 2C and D).

**Fig. 3:**
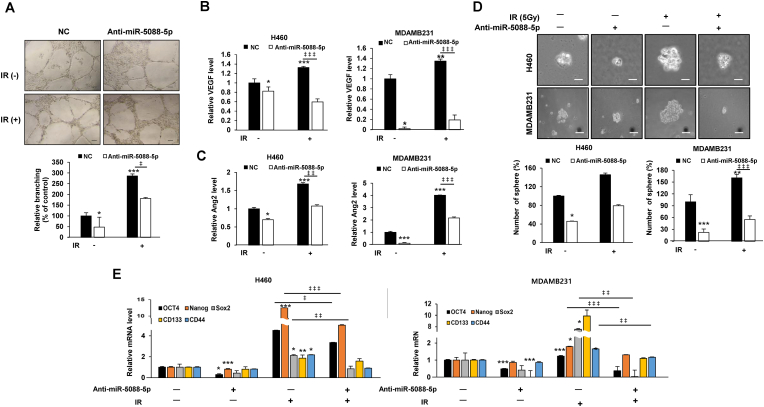
The miR-5088-5p inhibitor decreases the IR-induced angiogenesis and stemness maintenance. (A) Tube formation assay of angiogenic ability. After transfecting MDA-MB-231 cells with miR-5088-5p inhibitor or negative control (NC), supernatants were obtained with or without IR treatment. After seeding HUVECs (1 × 10^4^ cells/well) in a matrigel-coated 96-well plate, they were cultured for 16 h with each indicated supernatant. (Scale bar, 100 μm). The average number of capillary branches in six random fields was counted and graphed. (B–E) To determine the effect of miR-5088-5p on IR-induced angiogenesis and stemness maintenance, H460 and MDA-MB-231 cells were transfected with miR-5088-5p inhibitor or negative control and treated with/without IR (5Gy). (B) and (C) qRT-PCR analysis of expression of angiogenesis-related factors including VEGF (B) and Ang2 (C). (D) For sphere formation assay to confirm stemness, miR-5088-5p and IR combined cells were seeded in 100 mm culture dishes (1 × 10^5^ cells per dish) and cultured for 12 days. This experiment was performed in triplicate and the average number of colonies was plotted (Scale bar, 250 ㎛). (E) qRT-PCR analysis of expression of cancer stem-like cell markers, such as Oct4, Nanog, Sox2, CD133, and CD44, normalized to that of *U6.* Data are presented as mean ± S.D. *P* values are *P < 0.05, **P < 0.01, and ***P < 0.001 compared to control; ‡*P* < 0.05, ‡ ‡*P* < 0.01, and ‡ ‡ ‡*P* < 0.001 compared to IR group. Student's *t*-test.

Vascular endothelial growth factor (VEGF) is a key mediator of angiogenesis. It binds to two VEGF receptors (VEGF receptor-1 and VEGF receptor-2) expressed in vascular endothelial cells to proliferate and create new blood vessels [34,35]. In addition, angiopoietin 2 (Ang2), a member of the angiopoietin (Ang) family, is known to be involved in the formation of new blood vessels by binding to the TEK (Tie2) tyrosine kinase receptor of vascular endothelial cells to increase cell proliferation and migration [36,37]. Therefore, as a method for confirming the angiogenic ability, the tube formation assay and the mRNA expression of ang2 and VEGF, which are angiogenic markers, were confirmed. As a result, the miR-5088-5p inhibitor induced a decrease in IR-induced tube formation capacity (Fig. 3A) by inhibiting angiogenesis-related factors such as vascular endothelial growth factor (VEGF) and angiopoietin (Ang) 2 (Fig. 3B and C).

The sphere formation assay is a well-known experimental method used to identify cancer stem-like cells with self-renewing and pluripotent capabilities in solid tumors [38]. Also, OCT4, Sox2, Nanog, CD44 and CD133 are generally known as cancer stem-like cell markers [[39], [40], [41]]. To confirm the effect of miR-5088-5p, which was increased by IR, on stemness, we checked the expression of cancer stem-like cell markers and sphere formation ability. As a result, the miR-5088-5p inhibitor decreased in IR-induced sphere formation ability (Fig. 3D) by reducing cancer stem-like cell (CSC) marker proteins, such as Oct4, Nanog, Sox2, CD133, and CD44 (Fig. 3E). In addition, non-metastatic MCF-7 cells obtained the same results as H460 and MDA-MB-231 cells, reconfirming the mechanisms of radiation and miR-5088-5p inhibitors (Supplementary Fig. S5). The collective results clearly demonstrate that IR-induced miR-5088-5p enhances epithelial-mesenchymal transition (EMT), migration, invasion, angiogenesis, and stemness maintenance.

### 3.3 The miR-5088-5p inhibitor reduces IR-induced pulmonary metastasis

**Fig. 4:**
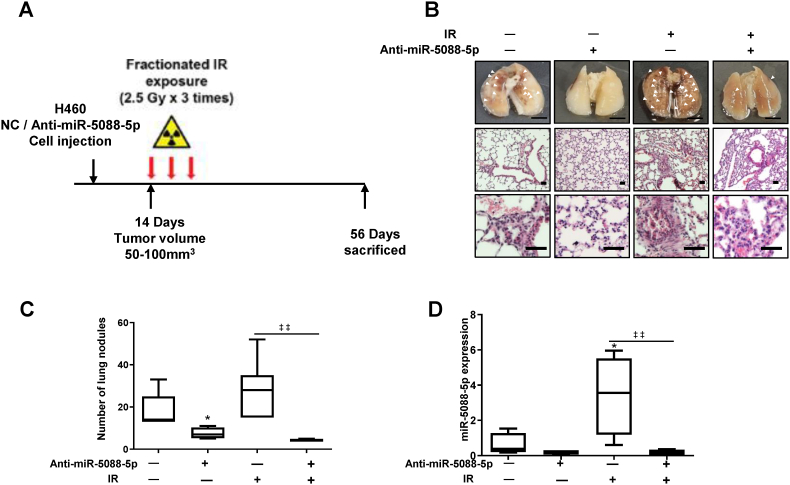
The miR-5088-5p inhibitor reduces IR-induced pulmonary metastasis. (A) For the animal experiment, H460 cells (2 × 10^6^ cells/mouse) transfected with the miR-5088-5p inhibitor were orthotopically injected into tail veins of nude mice (NC, n = 5; anti-miR-5088-5p, n = 4; NC + IR, n = 6; anti-miR-5088-5p + IR, n = 4). On day 14 of injection, mice were treated three times with IR (2.5Gy/day) and ultimately sacrificed at 56 days. (B) Images of pulmonary metastatic tissues were obtained (Scale bar, 500 mm) and stained with H&E (Scale bar, 500 μm). (C) The number of metastatic lung nodules was counted in xenograft mice. (D) qRT-PCR analysis of miR-5088-5p expression in plasma of the indicated groups of mice. (NC, n = 4; anti-miR-5088-5p, n = 3; NC + IR, n = 5; anti-miR-5088-5p + IR, n = 4). qRT-PCR data were quantified with *U6*. The data are reported as the mean ± S.D. *P* values are *P < 0.05 compared to control; ‡ ‡*P* < 0.01 compared to IR group. Student's *t*-test.

To validate the relevance of miR-5088-5p in the mechanism of IR-induced metastasis, a mouse xenograft model using H460 cells transfected with miR-5088-5p inhibitor (anti-miR-5088-5p) was employed. Six-week-old BALB/C nude mice were injected with H460 cells transfected with miR-5088-5p inhibitor into the tail vein. After 14 days, mice were subjected to IR (2.5 Gy/day) in the chest for 3 days (Fig. 4A). The radiation dose to induce malignancy was selected by referring to several previous papers [[42], [43], [44], [45]]. Histogram and H&E staining results showed that the lung metastasis pattern of the group of mice treated with radiation after injection of cells transfected cells with miR-5088-5p inhibitor was further reduced relative to the group exposed to radiation alone (Fig. 4B). The number of nodules in lung was counted and graphs generated (Fig. 4C). Based on the collective findings, we concluded that the miR-5088-5p inhibitor could effectively suppress IR-induced lung metastasis.

MiRNAs are important circulating factor in blood and extensive studies have focused on their potential as biomarkers for tumor diagnosis and treatment [[46], [47], [48]]. In our experiments, expression of miR-5088-5p was examined in plasma of mice, with a view to determining its potential as a diagnostic marker. Comparison of the levels of miR-5088-5p in each group revealed the most significant increase in IR-treated mice and decrease in mice co-treated with IR and miR-5088-5p inhibitor (Fig. 4D), supporting its clinical applicability as a biomarker for cancer metastasis. Thus, inhibition of miR-5088-5p presents an effective strategy to suppress metastasis due to radiation resistance and may be concomitantly utilized to improve the efficiency of radiotherapy.

### 3.4 The miR-5088-5p inhibitor reduces resistance to IR and drug therapy

Since surgery, radiotherapy, and chemotherapeutic drugs constitute common cancer treatments, the effects of miR-5088-5p inhibitor on cancer cell sensitivity to radiation and anticancer drug cisplatin were examined via colony formation and methylthiazole tetrazolium (MTT) analyses, respectively. In H460 and MDA-MB-231 cells transfected with the miR-5088-5p inhibitor, colony forming ability (Supplementary Fig. S6A) and cancer cell proliferation (Supplementary Fig. S6B) were reduced compared to the corresponding control group. Additionally, the proliferation rate was reduced in cells co-treated with miR-5088-5p inhibitor and radiation/cisplatin relative to those exposed to radiation/anticancer drug alone (Supplementary Figs. S6B and C). Our findings clearly suggest that miR-5088-5p inhibitor effectively promotes cancer cell death by increasing sensitivity to radiation and chemotherapy drugs.

### 3.5 The miR-5088-5p inhibitor attenuates migratory ability, invasiveness, and stemness maintenance via suppression of Slug

In order to discover a major factor involved in the suppression mechanism of miR-5088-5p inhibitors against IR-induced malignancy, mRNA expression of malignancy-related factors was compared using siRNA of Slug, Snail, and Zeb1 (Fig. 2A), which are transcription factors for EMT markers increased by IR. As a result, siRNA against Slug were most effectively reduced the mRNA expression of Vimentin and N-cadherin as EMT markers, CD44 as a stemness marker, VEGF as an angiogenesis marker, and MMP9 as an invasion-related marker MMP-9 (Supplementary Fig. S7). Therefore, it was expected that Slug acts as a key factor in the inhibitory mechanism of IR-induced malignancy by miR-5088-5p inhibitor.

**Fig. 5:**
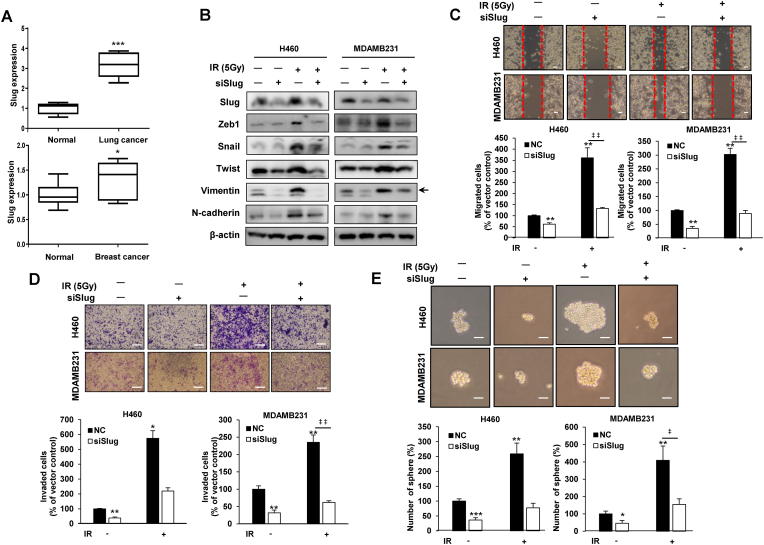
Slug act as a key factor in suppression of IR-induced malignancy by miR-5088-5p inhibitor. (A) Using the Oncomine public database (www.oncomine.org; lung (Wachi et al., 2005) and breast (Karnoub et al., 2007) cancers), expression of Slug in tissues of lung and breast cancer patients was determined. (B–E) H460 and MDA-MB-231 cells were transfected with siRNA against Slug or negative control (NC) and treated with or without IR (5 Gy). (B) Expression of EMT markers, Zeb1, Snail, Twist, Vimentin, and N-cadherin was confirmed 48 h after transfection by immunoblot analysis. β-actin was used as loading control. (C–E) Migratory ability (C) and invasiveness (D) were confirmed using wound healing (Scale bar, 100 ㎛) and transwell invasion assays (Scale bar, 100 ㎛), respectively, and stemness maintenance was tested using sphere formation assay (E) (Scale bar, 250 ㎛). The data are reported as the mean ± S.D. *P* values are *P < 0.05, **P < 0.001, and ***P < 0.001 compared to control; ‡*P* < 0.05 and ‡ ‡*P* < 0.01 compared to IR group. Student's *t*-test.

In addition, data from the available public dataset showed high expression of Slug in tumor tissues from lung and breast cancer patients relative to their normal tissue counterparts (Fig. 5A). To investigate the effect of Slug on IR-induced malignancy, we examined the expression of mesenchymal marker proteins, migration, invasion, and sphere formation assay using siRNA against Slug. As a result, IR-induced EMT markers such as Zeb1, Snail, Twist, Vimentin and N-cadherin were reduced by Slug siRNA (Fig. 5B). In addition, IR-induced increased migration, invasion, and sphere formation ability were all reduced by siSlug. (Fig. 5C–E). On the other hand, as a result of confirming the involvement of siSnail and siZeb1 in IR-induced malignancy, IR-induced cell migration, invasiveness, and stemness maintenance were reduced by siSnail and siZeb1 (Supplementary Fig. S8), but the efficacy by siSlug was the greatest (Fig. 5C–E). These results support a role for Slug as a key factor in IR-induced malignancy mechanisms. These results showed that reduction of Slug dramatically decreased IR-induced malignancy such as migration, invasion, stemness, and expression of theirs related factors.

**Fig. 6:**
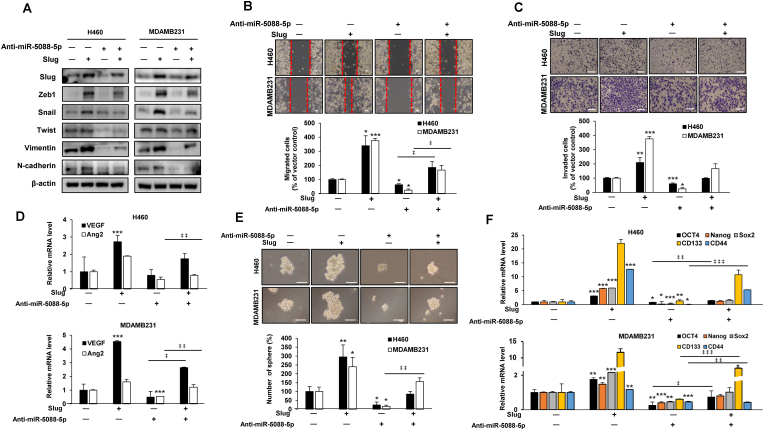
The miR-5088-5p inhibitor reduces malignant activity by suppressing Slug. (A–F) H460 and MDA-MB-231 cells were transfected with/without a Slug-overexpressing vector in the presence or absence of miR-5088-5p inhibitor and expression of mesenchymal-related proteins (A), migratory (B) and invasive (C) abilities, and angiogenesis-related factors (D) examined by immunoblot analysis, wound healing (Scale bar, 100㎛), transwell invasion (Scale bar, 100㎛), and qRT-PCR assays, respectively. β-actin was used as loading control for immunoblot analysis. (E–F) Measurement of stemness maintenance by the sphere formation assay (Scale bar, 250㎛) (E) and mRNA expression of cancer stem-like cell markers (F). qRT-PCR data was quantified with *U6*. The data are presented as the mean ± S.D. *P* values are *P < 0.05, **P < 0.01, and ***P < 0.001 compared to control; ‡*P* < 0.05, ‡ ‡*P* < 0.01, and ‡ ‡ ‡*P* < 0.001 compared to anti-miR-5088-5p group. Student's *t*-test.

**Fig. 7:**
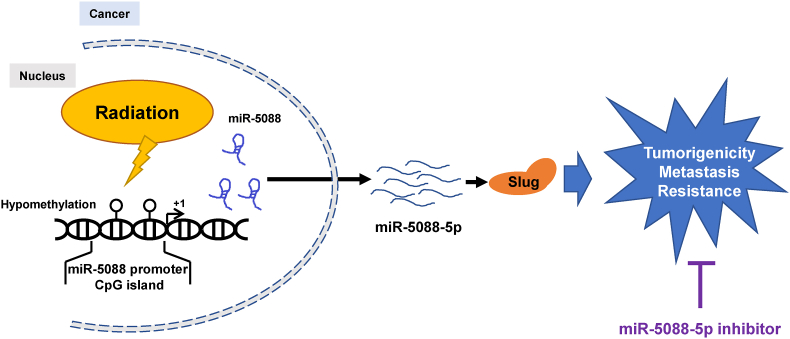
Schematic diagram showing that the miR-5088-5p inhibitor suppresses IR-induced tumor malignancy through reduction of Slug expression. The miR-5088-5p inhibitor suppresses IR-induced tumor tumorigenicity, metastasis, and resistance to IR and anticancer drug.

Next, we tested the involvement of Slug in the inhibitory mechanism of IR-induced malignancy by miR-5088-5p inhibitor using miR-5088-5p inhibitor and Slug overexpressing vector. As a result, the miR-5088-5p inhibitor effectively reduced Slug-induced mesenchymal trait (Fig. 6A), migration capacity (Fig. 6B), invasiveness (Fig. 6C), the expression of angiogenesis-related factors, VEGF and Ang2 (Fig. 6D), sphere formation ability (Fig. 6E), and mRNA levels of the cancer stem-like cell markers Oct4, Nanog, Sox2, CD133, and CD44 (Fig. 6F). Accordingly, we propose that the miR-5088-5p inhibitor decreases IR-induced tumorigenicity through suppression of the miR-5088-5p-Slug signaling pathway (Fig. 7).

## 4 Discussion

Ionizing radiation (IR) is a commonly used effective therapeutic tool for cancer [49,50]. However, cancer cells that survive after radiotherapy can develop malignant characteristics, including EMT and stemness [[51], [52], [53], [54]], promoting tumor recurrence, and metastasis [51,55,56]. Therefore, considerable research efforts to reduce the side-effects triggered by radiation are underway to enhance treatment efficiency. Here, we focused on miRNAs as a tool to understand the mechanisms underlying radiation-induced malignancy and optimize the therapeutic effects of radiation.

MiRNA regulate the post-transcriptional expression of target mRNA molecules and are involved in various biological processes and diseases [[46], [47], [48]]. Since miRNAs are differentially expressed in various diseases and targets multiple mRNAs, their utility as novel therapeutic targets is a hot topic of interest [57]. At present, small-interfering RNA (siRNA) approved by the FDA in 2018 as a therapeutic small RNA molecules [58,59] and miRNAs (including miR-34 (MRX34), miR-92 (MRG 110), miR-16 (MesomiR-1), miR-122 (Miravirsen), miR-29 (MRG-201), miR-21 (RG-012), and miR-155 (Cobomarsen-MRG-106)) are in clinical trials for various diseases [59,60]. Studies are further underway to transform miRNA into stable forms for use as therapeutic agents for diseases [61,62].

Previously, we showed that miR-5088-5p is more highly expressed in breast cancer patients and increases malignancy and metastasis via downregulating target DBC2 (Deleted in Breast Cancer 2; RHOBTB2) [25]. Since overexpression of miR-5088-5p has a positive relationship with tumorigenicity and metastasis in breast cancer patients, the expression pattern of miR-5088-5p in plasma showed potential as a target for diagnosing tumor malignancy. In this current study, a correlation between radiation-induced malignancy and overexpression of miR-5088-5p as an onco-miRNA in lung and breast cancer was established. The expression levels of miR-5088-5p were increased in both animal models (Fig. 1B) and cells (Fig. 1C) as well as breast and lung cancer patients subjected to radiotherapy (Fig. 1A), Supplementary Fig. S1) while levels of target DBC2 were relatively decreased (Supplementary Fig. S2), supporting our earlier findings.

Additionally, to analyze whether change in miR-5088-5p expression under conditions of radiation occur as a result of epigenetic modification, methylation of the miR-5088-5p promoter was assessed. IR induced hypomethylation of the miR-5088-5p promoter through suppression of DNMT1, DNMT3a, and DNMT3b *in vivo* and in vitro (Fig. 1D–F), Supplementary Figs. S3–4), consistent with earlier reports that radiation induces DNA hypomethylation [25].

To elucidate the specific mechanisms underlying upregulation of miR-5088-5p upon radiation treatment in lung and breast cancer cells, a miR-5088-5p inhibitor was used. At the cellular level, miR-5088-5p inhibitor reduced IR-induced EMT, migration, invasion, angiogenesis, and stemness maintenance (Fig. 2, Fig. 3) by decreasing Slug, and increased sensitivity to radiation and cisplatin, thereby decreasing cell viability (Supplementary Fig. S6). Additionally, in an experimental animal model, IR-induced lung metastasis was reduced by miR-5088-5p inhibitor (Fig. 4), supporting the results at the cellular level (Fig. 2, Fig. 3). Slug is overexpressed in several carcinomas including ovarian cancer, lung cancer, breast cancer, and gastric cancer and is associated with poor prognosis of patient [65–68], and has been reported as a radioresistance factor in glioblastoma, cholangiocarcinoma, and colorectal cancer [69–71]. Previous reports support the finding that miR-5088-5p, which is increased by radiation, induces malignancy by increasing Slug.

Taken together, this study showed the potential as a new targeted therapeutic agent that can increase the efficiency of radiation therapy when the miR-5088-5p inhibitor is used in combination with radiotherapy.

## 5 Conclusion

Radiation is a well-known method of treating many cancers, but it is known that cancer cells that survive after radiotherapy cause recurrence and metastasis. In order to increase the effectiveness of radiotherapy, a combination therapy with radiation to suppress radiation-induced malignancy is absolutely necessary. In this paper, we confirm that the miR-5088-5p-Slug axis is mainly involved in radiation-induced malignancy and that miR-5088-5p inhibitors decreased the mechanism of radiation-induced malignancy. These results show the potential of miR-5088-5p inhibitors as a combination therapy to improve the effectiveness of radiotherapy.

## Funding

This study was supported by grants of the 10.13039/501100003725National Research Foundation of Korea and the 10.13039/501100008003Korea Institute of Radiological and Medical Sciences (KIRAMS), funded by Ministry of Science and ICT (MSIT) in Republic of Korea [No. NRF-2021R1A2C2005966 (50698-2022) and 50531-2023].

## CRediT authorship contribution statement

**Hyun Jeong Seok:** designed the experiments and drafted the manuscript. designed and performed animal experiments. designed and performed MSP and qMSP. All authors discussed the results and commented on the manuscript. **Jae Yeon Choi:** designed and performed animal experiments. All authors discussed the results and commented on the manuscript. **Joo Mi Yi:** designed and performed MSP and qMSP. All authors discussed the results and commented on the manuscript. **In Hwa Bae:** supervised the work, designed the experiments and drafted the manuscript. designed and performed animal experiments. designed and performed MSP and qMSP. All authors discussed the results and commented on the manuscript.

## Declaration of competing interest

The authors declare no potential conflicts of interest.
