# Supplementary material for: Targeting miR-5088-5p attenuates radioresistance by suppressing Slug
Source: Noncoding RNA Res. 2023 Jan 2;8(2):164–73. doi: 10.1016/j.ncrna.2022.12.005 (PMC9827365; doi:10.1016/j.ncrna.2022.12.005)
Supplement: Multimedia component 1 [file mmc1.pptx]

## Slide 1
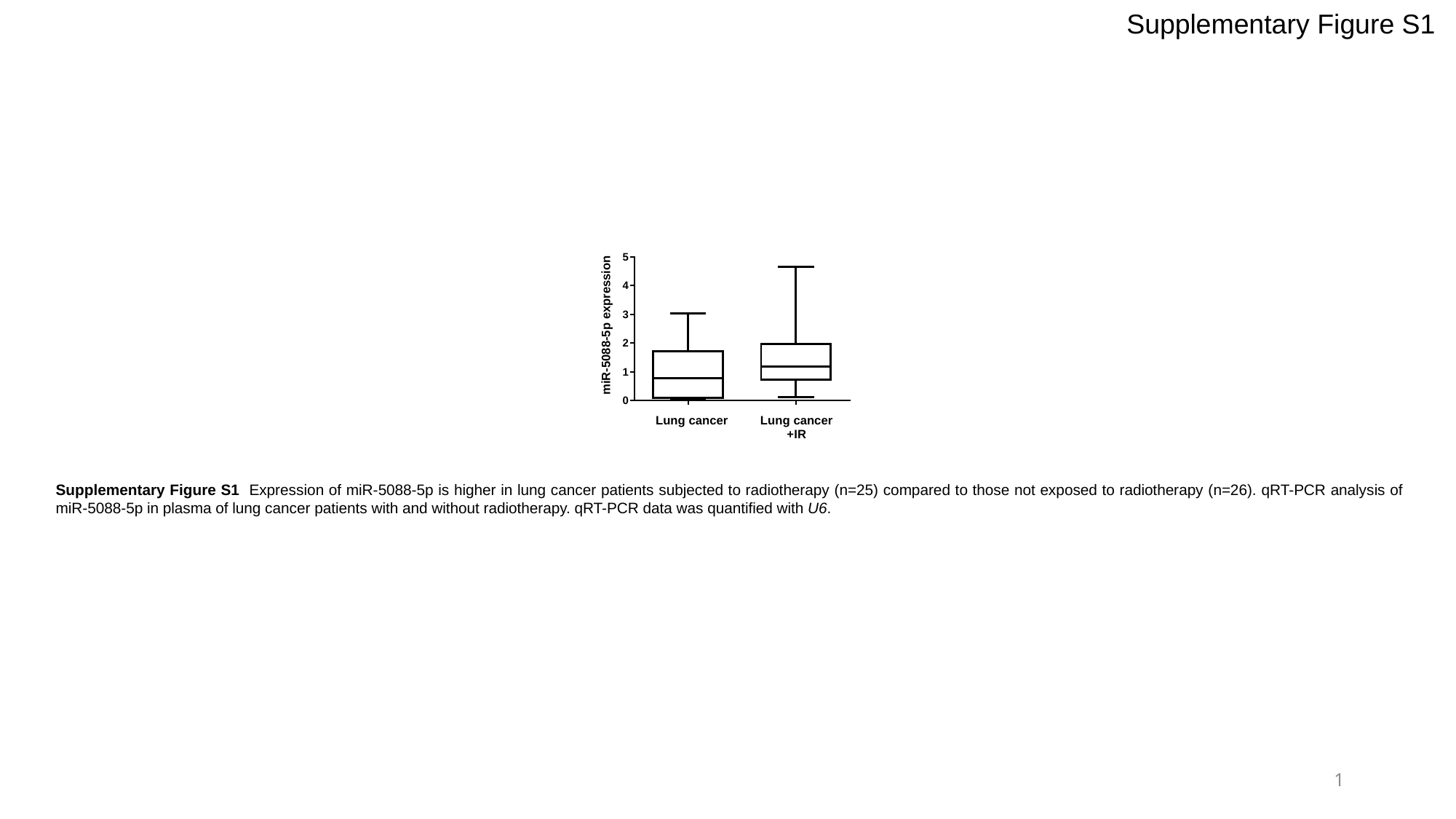

Supplementary Figure S1
miR-5088-5p expression
Lung cancer
Lung cancer
+IR
Supplementary Figure S1 Expression of miR-5088-5p is higher in lung cancer patients subjected to radiotherapy (n=25) compared to those not exposed to radiotherapy (n=26). qRT-PCR analysis of miR-5088-5p in plasma of lung cancer patients with and without radiotherapy. qRT-PCR data was quantified with U6.
1

## Slide 2
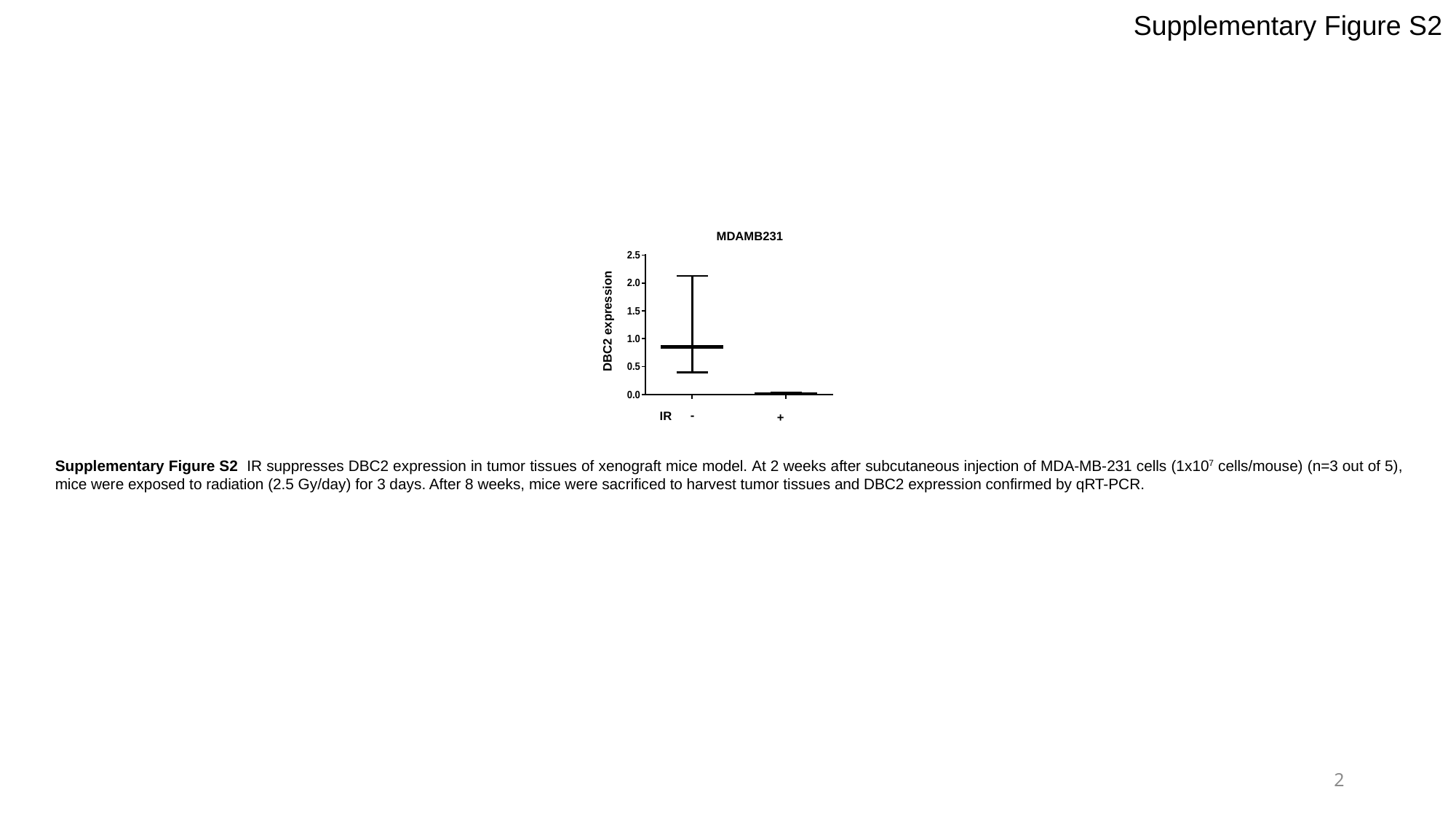

Supplementary Figure S2
MDAMB231
DBC2 expression
-
IR
+
Supplementary Figure S2 IR suppresses DBC2 expression in tumor tissues of xenograft mice model. At 2 weeks after subcutaneous injection of MDA-MB-231 cells (1x10^7^ cells/mouse) (n=3 out of 5), mice were exposed to radiation (2.5 Gy/day) for 3 days. After 8 weeks, mice were sacrificed to harvest tumor tissues and DBC2 expression confirmed by qRT-PCR.
2

## Slide 3
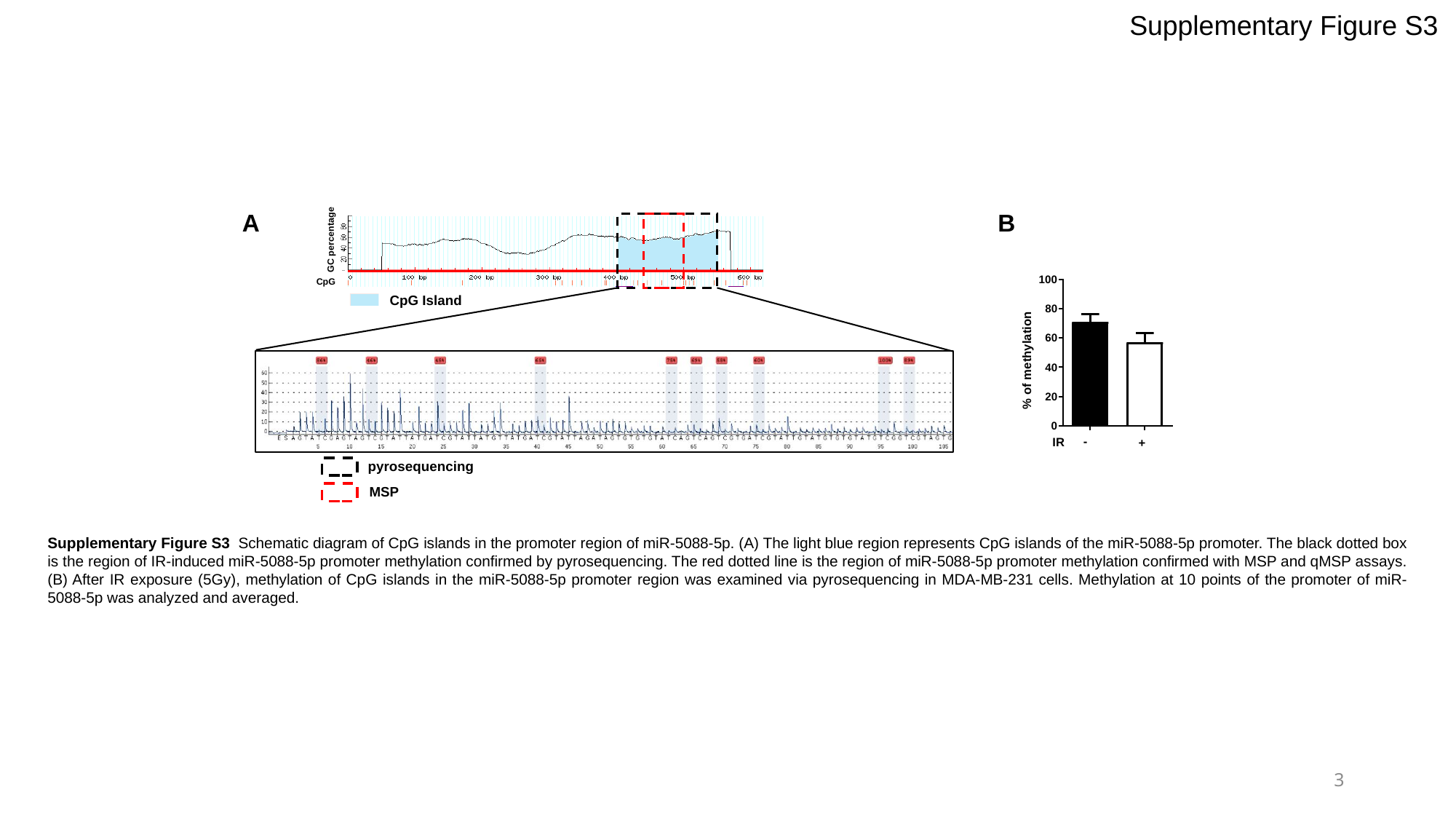

Supplementary Figure S3
A
B
pyrosequencing
MSP
GC percentage
% of methylation
-
IR
+
CpG
CpG Island
Supplementary Figure S3 Schematic diagram of CpG islands in the promoter region of miR-5088-5p. (A) The light blue region represents CpG islands of the miR-5088-5p promoter. The black dotted box is the region of IR-induced miR-5088-5p promoter methylation confirmed by pyrosequencing. The red dotted line is the region of miR-5088-5p promoter methylation confirmed with MSP and qMSP assays. (B) After IR exposure (5Gy), methylation of CpG islands in the miR-5088-5p promoter region was examined via pyrosequencing in MDA-MB-231 cells. Methylation at 10 points of the promoter of miR-5088-5p was analyzed and averaged.
3

## Slide 4
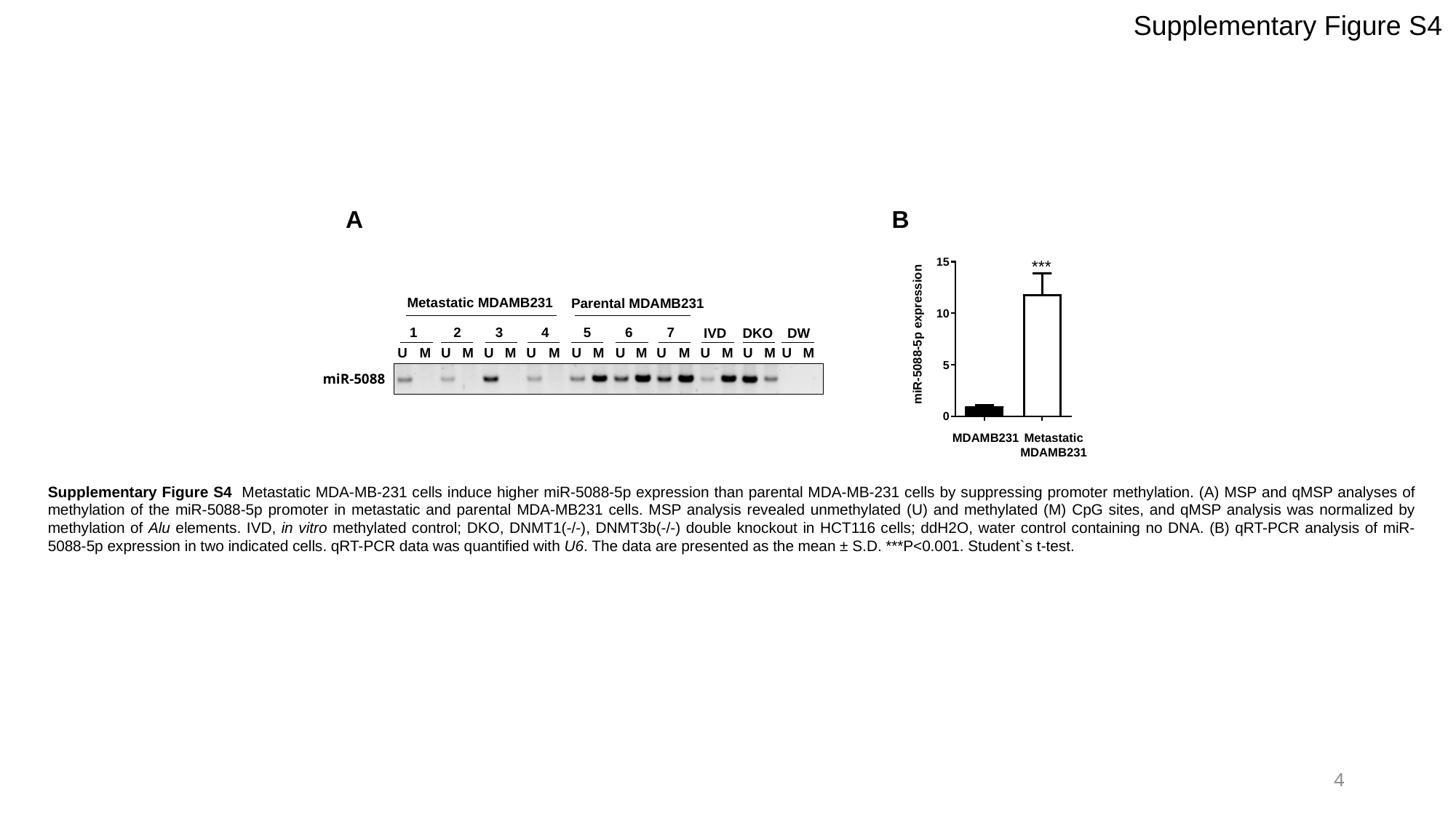

Supplementary Figure S4
A
B
***
miR-5088-5p expression
MDAMB231
Metastatic
MDAMB231
Metastatic MDAMB231
Parental MDAMB231
1
2
3
4
5
6
7
IVD
DKO
DW
U
M
U
M
U
M
U
M
U
M
U
M
U
M
U
M
U
M
U
M
miR-5088
Supplementary Figure S4 Metastatic MDA-MB-231 cells induce higher miR-5088-5p expression than parental MDA-MB-231 cells by suppressing promoter methylation. (A) MSP and qMSP analyses of methylation of the miR-5088-5p promoter in metastatic and parental MDA-MB231 cells. MSP analysis revealed unmethylated (U) and methylated (M) CpG sites, and qMSP analysis was normalized by methylation of Alu elements. IVD, in vitro methylated control; DKO, DNMT1(-/-), DNMT3b(-/-) double knockout in HCT116 cells; ddH2O, water control containing no DNA. (B) qRT-PCR analysis of miR-5088-5p expression in two indicated cells. qRT-PCR data was quantified with U6. The data are presented as the mean ± S.D. ***P<0.001. Student`s t-test.
4

## Slide 5
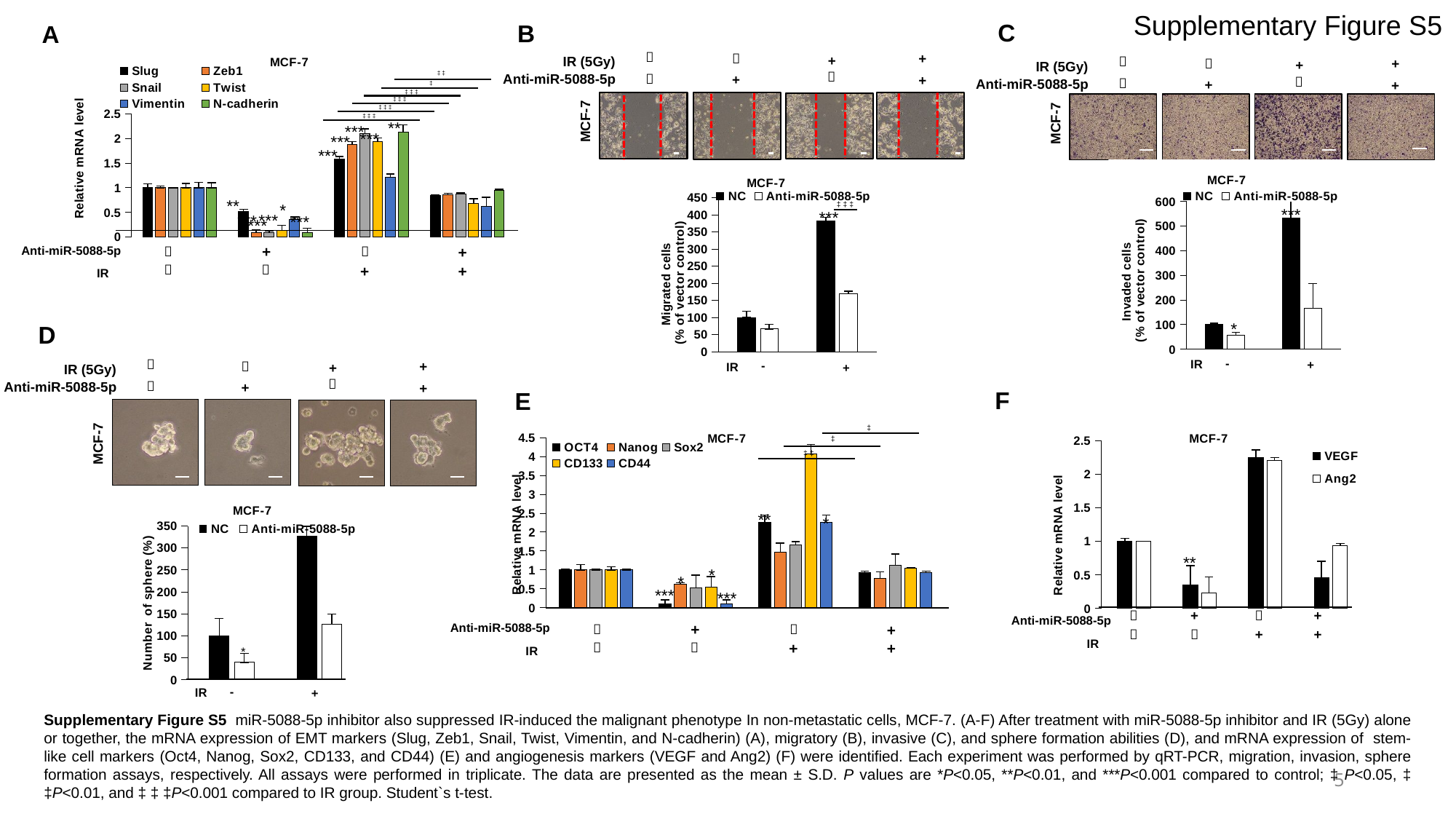

Supplementary Figure S5
C
B
A
### Chart: MCF-7
| Category | Slug | Zeb1 | Snail | Twist | Vimentin | N-cadherin |
|---|---|---|---|---|---|---|
| NC | 1.0 | 1.0 | 1.0 | 1.0 | 1.0 | 1.0 |
| anti-miR-5088-5p | 0.5156950359001824 | 0.09629396405848888 | 0.08884433950481116 | 0.13657719223290007 | 0.3630100025333881 | 0.08991850432710277 |
| NC | 1.5888739508487264 | 1.8720383459255383 | 2.1006572369814043 | 1.931293684528208 | 1.2113392134552392 | 2.132381311055557 |
| anti-miR-5088-5p | 0.8412094503143782 | 0.8623320144985892 | 0.8786710048627586 | 0.6877726059108585 | 0.628200172784563 | 0.9411681383781197 |ㅡ
ㅡ
+
+
IR (5Gy)
ㅡ
ㅡ
Anti-miR-5088-5p
+
+
MCF-7
ㅡ
ㅡ
+
+
IR (5Gy)
ㅡ
ㅡ
Anti-miR-5088-5p
+
+
MCF-7
B
‡ ‡
‡
‡ ‡ ‡
‡ ‡ ‡
‡ ‡ ‡
migration
‡ ‡ ‡
**
***
***
***
***
### Chart: MCF-7
| Category | NC | Anti-miR-5088-5p |
|---|---|---|
| ㅡ | 100.0 | 57.06214689 |
| IR | 533.0508475 | 166.1016949 |***
*
-
IR
+
### Chart: MCF-7
| Category | NC | Anti-miR-5088-5p |
|---|---|---|
| ㅡ | 100.0 | 66.49831649831648 |
| IR | 381.64983164983164 | 170.03367003367003 |**
‡ ‡ ‡
*
***
***
***
***
***
+
Anti-miR-5088-5p
ㅡ
ㅡ
+
ㅡ
ㅡ
+
+
IR
D
ㅡ
ㅡ
+
+
IR (5Gy)
ㅡ
ㅡ
Anti-miR-5088-5p
+
+
MCF-7
-
IR
+
F
E
‡
### Chart: MCF-7
| Category | VEGF | Ang2 |
|---|---|---|
| NC | 1.0 | 1.0 |
| anti-miR-5088-5p | 0.3530480431738337 | 0.22615271171275622 |
| NC | 2.2498441257998105 | 2.206466623666173 |
| anti-miR-5088-5p | 0.4588960179146345 | 0.9334544204614091 |
### Chart: MCF-7
| Category | OCT4 | Nanog | Sox2 | CD133 | CD44 |
|---|---|---|---|---|---|
| NC | 1.0 | 1.0 | 1.0 | 1.0 | 1.0 |
| anti-miR-5088-5p | 0.10219194246257329 | 0.6119867032773271 | 0.5300682804263783 | 0.5428311196106347 | 0.10219194246257329 |
| NC | 2.256578334746616 | 1.4610508900666435 | 1.6522738345838768 | 4.076678630009703 | 2.256578334746616 |
| anti-miR-5088-5p | 0.9358203035126914 | 0.7676962680637245 | 1.115500458025729 | 1.0522066684327807 | 0.9358203035126914 |**
*
*
*
***
***
+
Anti-miR-5088-5p
ㅡ
ㅡ
+
ㅡ
ㅡ
+
+
IR
‡
‡ ‡
### Chart: MCF-7
| Category | NC | Anti-miR-5088-5p |
|---|---|---|
| ㅡ | 100.0 | 40.0 |
| IR | 326.6666666666667 | 126.66666666666666 |*
-
IR
+
**
ㅡ
+
ㅡ
+
Anti-miR-5088-5p
ㅡ
ㅡ
+
+
IR
Supplementary Figure S5 miR-5088-5p inhibitor also suppressed IR-induced the malignant phenotype In non-metastatic cells, MCF-7. (A-F) After treatment with miR-5088-5p inhibitor and IR (5Gy) alone or together, the mRNA expression of EMT markers (Slug, Zeb1, Snail, Twist, Vimentin, and N-cadherin) (A), migratory (B), invasive (C), and sphere formation abilities (D), and mRNA expression of stem-like cell markers (Oct4, Nanog, Sox2, CD133, and CD44) (E) and angiogenesis markers (VEGF and Ang2) (F) were identified. Each experiment was performed by qRT-PCR, migration, invasion, sphere formation assays, respectively. All assays were performed in triplicate. The data are presented as the mean ± S.D. P values are *P<0.05, **P<0.01, and ***P<0.001 compared to control; ‡ P<0.05, ‡ ‡P<0.01, and ‡ ‡ ‡P<0.001 compared to IR group. Student`s t-test.
5

## Slide 6
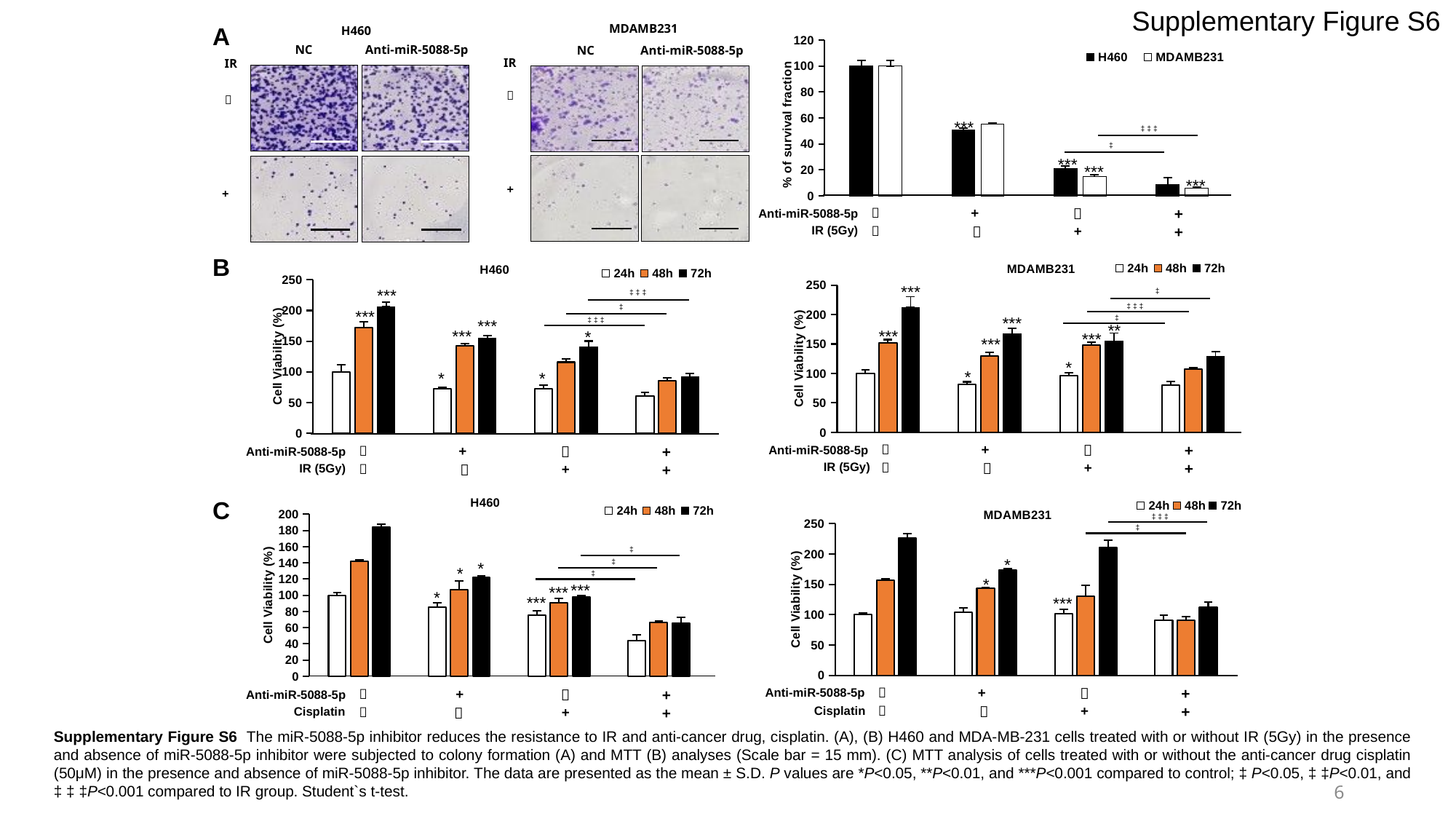

Supplementary Figure S6
MDAMB231
A
### Chart
| Category | H460 | MDAMB231 |
|---|---|---|
| NC | 100.0 | 100.0 |
| Anti-miR-5088-5p | 50.57915057915058 | 55.05952380952381 |
| NC | 21.235521235521233 | 15.178571428571427 |
| Anti-miR-5088-5p | 8.880308880308881 | 5.952380952380952 |ㅡ
+
ㅡ
+
Anti-miR-5088-5p
IR (5Gy)
ㅡ
ㅡ
+
+
H460
NC
Anti-miR-5088-5p
NC
Anti-miR-5088-5p
IR
IR
ㅡ
ㅡ
***
‡ ‡ ‡
‡
***
***
***
+
+
B
### Chart: MDAMB231
| Category | 24h | 48h | 72h |
|---|---|---|---|
| NC | 100.0 | 151.27375321550082 | 212.19815782922575 |
| Anti-miR-5088-5p | 81.1799850634802 | 129.10131939258153 | 167.09816612729233 |
| NC | 96.12480292091941 | 148.60177578624177 | 154.36893203883494 |
| Anti-miR-5088-5p | 80.15932287776947 | 107.03676043481869 | 129.09302132603105 |***
***
**
***
***
***
*
*
ㅡ
+
ㅡ
+
Anti-miR-5088-5p
IR (5Gy)
ㅡ
ㅡ
+
+
### Chart: H460
| Category | 24h | 48h | 72h |
|---|---|---|---|
| NC | 100.0 | 172.5640878591501 | 206.5206170786259 |
| Anti-miR-5088-5p | 72.27734128577164 | 141.88220450765613 | 155.07828181453232 |
| NC | 71.83001663130125 | 115.72518208407409 | 140.47141136663416 |
| Anti-miR-5088-5p | 60.4232379423066 | 85.7888398233641 | 92.26357745024947 |***
***
***
***
*
*
*
ㅡ
+
ㅡ
+
Anti-miR-5088-5p
IR (5Gy)
ㅡ
ㅡ
+
+
‡
‡ ‡ ‡
‡ ‡ ‡
‡
‡
‡ ‡ ‡
C
### Chart: H460
| Category | 24h | 48h | 72h |
|---|---|---|---|
| NC | 100.0 | 141.68932038834953 | 184.2135922330097 |
| Anti-miR-5088-5p | 85.55339805825243 | 107.22330097087381 | 121.74757281553399 |
| NC | 75.10679611650485 | 90.37864077669904 | 97.96116504854369 |
| Anti-miR-5088-5p | 43.72815533980582 | 66.0485436893204 | 65.62135922330097 |*
*
***
***
*
***
ㅡ
+
ㅡ
+
Anti-miR-5088-5p
Cisplatin
ㅡ
+
+
ㅡ
### Chart: MDAMB231
| Category | 24h | 48h | 72h |
|---|---|---|---|
| NC | 100.0 | 156.80129990714948 | 225.88207985143916 |
| Anti-miR-5088-5p | 104.29433611884866 | 143.96471680594243 | 173.769730733519 |
| NC | 101.02135561745591 | 130.73351903435466 | 210.39925719591457 |
| Anti-miR-5088-5p | 90.34354688950789 | 90.50603528319404 | 112.58588672237697 |*
*
***
ㅡ
+
ㅡ
+
Anti-miR-5088-5p
ㅡ
ㅡ
+
+
‡ ‡ ‡
‡
‡
‡
‡
Cisplatin
Supplementary Figure S6 The miR-5088-5p inhibitor reduces the resistance to IR and anti-cancer drug, cisplatin. (A), (B) H460 and MDA-MB-231 cells treated with or without IR (5Gy) in the presence and absence of miR-5088-5p inhibitor were subjected to colony formation (A) and MTT (B) analyses (Scale bar = 15 mm). (C) MTT analysis of cells treated with or without the anti-cancer drug cisplatin (50μM) in the presence and absence of miR-5088-5p inhibitor. The data are presented as the mean ± S.D. P values are *P<0.05, **P<0.01, and ***P<0.001 compared to control; ‡ P<0.05, ‡ ‡P<0.01, and ‡ ‡ ‡P<0.001 compared to IR group. Student`s t-test.
6

## Slide 7
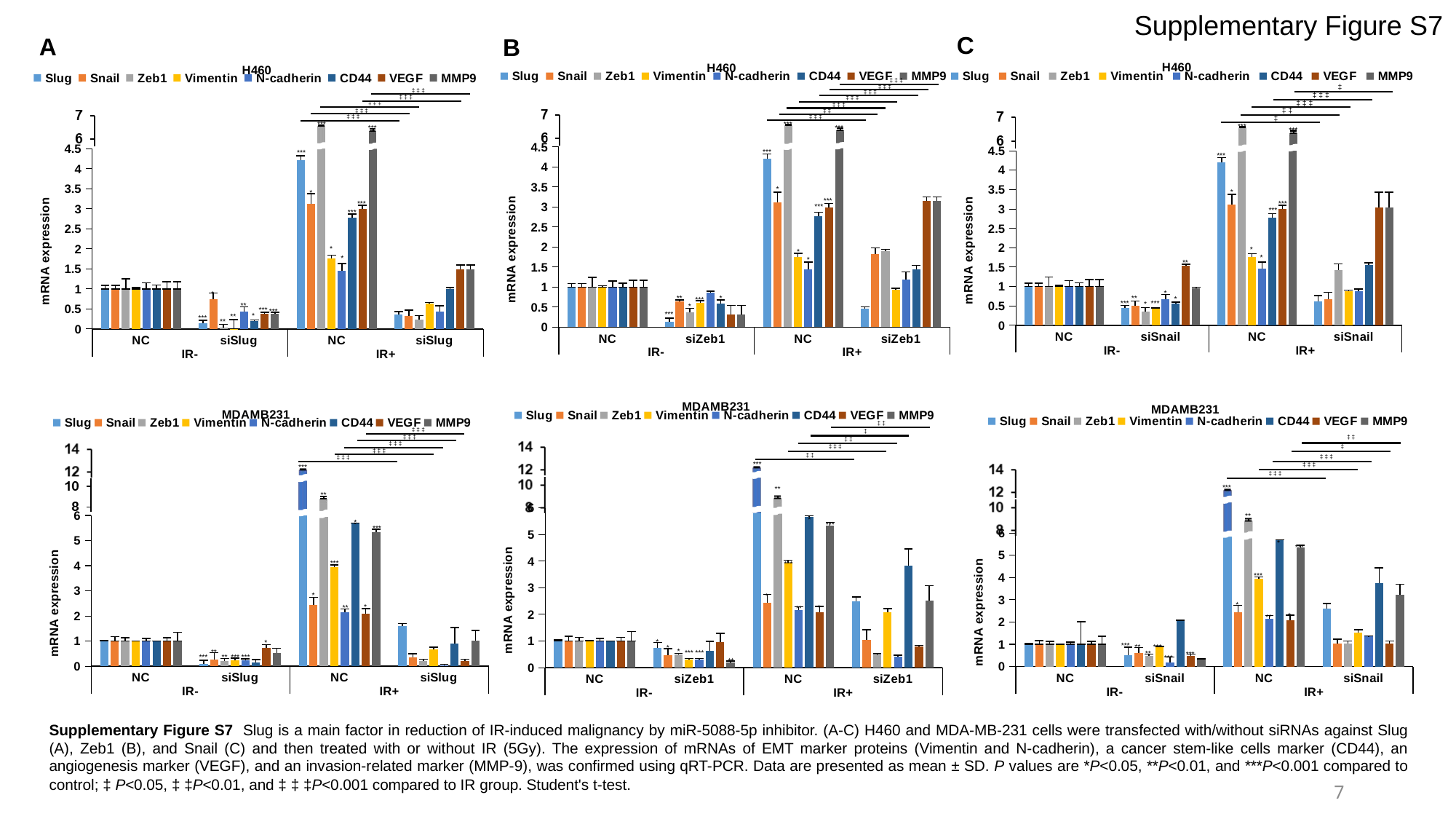

Supplementary Figure S7
C
A
B
### Chart: H460
| Category | Slug | Snail | Zeb1 | Vimentin | N-cadherin | CD44 | VEGF | MMP9 |
|---|---|---|---|---|---|---|---|---|
| NC | 1.0 | 1.0 | 1.0 | 1.0 | 1.0 | 1.0 | 1.0 | 1.0 |
| siSnail | 0.4551251293822219 | 0.5081200099623921 | 0.3521429365755296 | 0.4203893006898348 | 0.6783678362383048 | 0.5675985132631034 | 1.5332441242812727 | 0.9460056830723682 |
| NC | 4.206746055383941 | 3.1199162272643473 | 6.554546683284516 | 1.756946905545009 | 1.4513630646166242 | 2.771800136775429 | 2.9919126402866176 | 6.339642047861477 |
| siSnail | 0.6167961649194149 | 0.6643962019819236 | 1.4276969795934868 | 0.8845458186433447 | 0.8870050482302075 | 1.5553069403190003 | 3.0315058752755624 | 3.0315058752755624 |
### Chart: H460
| Category | Slug | Snail | Zeb1 | Vimentin | N-cadherin | CD44 | VEGF | MMP9 |
|---|---|---|---|---|---|---|---|---|
| NC | 1.0 | 1.0 | 1.0 | 1.0 | 1.0 | 1.0 | 1.0 | 1.0 |
| siZeb1 | 0.136451376806467 | 0.6460947333545766 | 0.36541444434783316 | 0.5979323518240671 | 0.8625715926895853 | 0.5877739531418048 | 0.32436434577781986 | 0.32436434577781986 |
| NC | 4.206746055383941 | 3.1199162272643473 | 6.554546683284516 | 1.756946905545009 | 1.4513630646166242 | 2.771800136775429 | 2.9919126402866176 | 6.339642047861477 |
| siZeb1 | 0.4582395203688057 | 1.8324637032372055 | 1.8960659509470013 | 0.939446488012034 | 1.1865048365409676 | 1.4380925925926644 | 3.1479254707441626 | 3.1479254707441626 |
### Chart: H460
| Category | Slug | Snail | Zeb1 | Vimentin | N-cadherin | CD44 | VEGF | MMP9 |
|---|---|---|---|---|---|---|---|---|
| NC | 1.0 | 1.0 | 1.0 | 1.0 | 1.0 | 1.0 | 1.0 | 1.0 |
| siSlug | 0.13730995530456547 | 0.7490331687599263 | 0.0292934548888662 | 0.0009054410344919835 | 0.42734495822414026 | 0.19430741954578978 | 0.3695153503242449 | 0.3695153503242449 |
| NC | 4.206746055383941 | 3.1199162272643473 | 6.554546683284516 | 1.756946905545009 | 1.4513630646166242 | 2.771800136775429 | 2.9919126402866176 | 6.339642047861477 |
| siSlug | 0.3630896623655969 | 0.32087956306475346 | 0.23068930132072382 | 0.6371603513064161 | 0.4283916606737065 | 0.9952651285861415 | 1.4796574525811639 | 1.4796574525811639 |‡ ‡ ‡
‡
‡ ‡ ‡
‡ ‡ ‡
‡ ‡ ‡
‡ ‡ ‡
‡ ‡ ‡
‡ ‡ ‡
‡ ‡ ‡
‡ ‡ ‡
‡ ‡ ‡
‡ ‡
‡ ‡ ‡
‡ ‡
‡ ‡ ‡
 ‡
‡ ‡ ‡
***
***
***
***
***
***
***
***
***
*
*
*
***
***
***
***
***
***
*
*
*
*
*
*
**
*
*
*
**
**
*
***
***
***
*
**
*
***
***
***
*
**
***
**
### Chart: MDAMB231
| Category | Slug | Snail | Zeb1 | Vimentin | N-cadherin | CD44 | VEGF | MMP9 |
|---|---|---|---|---|---|---|---|---|
| NC | 1.0 | 1.0 | 1.0 | 1.0 | 1.0 | 1.0 | 1.0 | 1.0 |
| siZeb1 | 0.7457045303204693 | 0.46005371105196097 | 0.48365983491164716 | 0.31577492204510366 | 0.30486533448956177 | 0.6185363750353267 | 0.9628600157356472 | 0.18246762203334524 |
| NC | 12.193193618673353 | 2.4236646684663214 | 8.820817351133131 | 3.930491212424835 | 2.1537463125197625 | 5.668398493120201 | 2.0814357286478873 | 5.341303431086662 |
| siZeb1 | 2.4965720429362586 | 1.030708715188978 | 0.46619218830546966 | 2.0880892528149704 | 0.41373506765408263 | 3.8345065332534225 | 0.7822292570614405 | 2.528637429630073 |
### Chart: MDAMB231
| Category | Slug | Snail | Zeb1 | Vimentin | N-cadherin | CD44 | VEGF | MMP9 |
|---|---|---|---|---|---|---|---|---|
| NC | 1.0 | 1.0 | 1.0 | 1.0 | 1.0 | 1.0 | 1.0 | 1.0 |
| siSnail | 0.5103099578295672 | 0.6232707743068495 | 0.4863886092762257 | 0.8828307585759884 | 0.18482415643087952 | 2.0425782671485178 | 0.4863886092762257 | 0.3405985874795135 |
| NC | 12.193193618673353 | 2.4236646684663214 | 8.820817351133131 | 3.930491212424835 | 2.1537463125197625 | 5.668398493120201 | 2.0814357286478873 | 5.341303431086662 |
| siSnail | 2.592876949642531 | 1.0385417605013076 | 1.026207646901816 | 1.530748740072835 | 1.3531690768103306 | 3.7489891465511334 | 1.026207646901816 | 3.219842777360247 |
### Chart: MDAMB231
| Category | Slug | Snail | Zeb1 | Vimentin | N-cadherin | CD44 | VEGF | MMP9 |
|---|---|---|---|---|---|---|---|---|
| NC | 1.0 | 1.0 | 1.0 | 1.0 | 1.0 | 1.0 | 1.0 | 1.0 |
| siSlug | 0.09064347350995655 | 0.2783944286732366 | 0.21475331211025078 | 0.22555400235754233 | 0.24211262358523256 | 0.15460320611701273 | 0.7339346542667233 | 0.5297228845927177 |
| NC | 12.193193618673353 | 2.4236646684663214 | 8.820817351133131 | 3.930491212424835 | 2.1537463125197625 | 5.668398493120201 | 2.0814357286478873 | 5.341303431086662 |
| siSlug | 1.5993067821774996 | 0.3646019356845032 | 0.21849803761010728 | 0.6601913753246527 | 0.03837381011921572 | 0.9098305460748574 | 0.21849803761010728 | 1.0201813866665306 |‡ ‡
‡ ‡ ‡
 ‡
‡ ‡ ‡
‡ ‡
‡ ‡
‡ ‡ ‡
‡ ‡ ‡
‡
‡ ‡ ‡
‡ ‡
‡ ‡ ‡
‡ ‡ ‡
***
‡ ‡ ‡
***
‡ ‡ ‡
***
**
**
**
*
*
***
***
*
***
***
***
***
*
*
*
*
*
**
**
*
**
*
*
***
***
**
*
*
**
***
***
**
***
***
**
***
***
***
**
Supplementary Figure S7 Slug is a main factor in reduction of IR-induced malignancy by miR-5088-5p inhibitor. (A-C) H460 and MDA-MB-231 cells were transfected with/without siRNAs against Slug (A), Zeb1 (B), and Snail (C) and then treated with or without IR (5Gy). The expression of mRNAs of EMT marker proteins (Vimentin and N-cadherin), a cancer stem-like cells marker (CD44), an angiogenesis marker (VEGF), and an invasion-related marker (MMP-9), was confirmed using qRT-PCR. Data are presented as mean ± SD. P values are *P<0.05, **P<0.01, and ***P<0.001 compared to control; ‡ P<0.05, ‡ ‡P<0.01, and ‡ ‡ ‡P<0.001 compared to IR group. Student's t-test.
7

## Slide 8
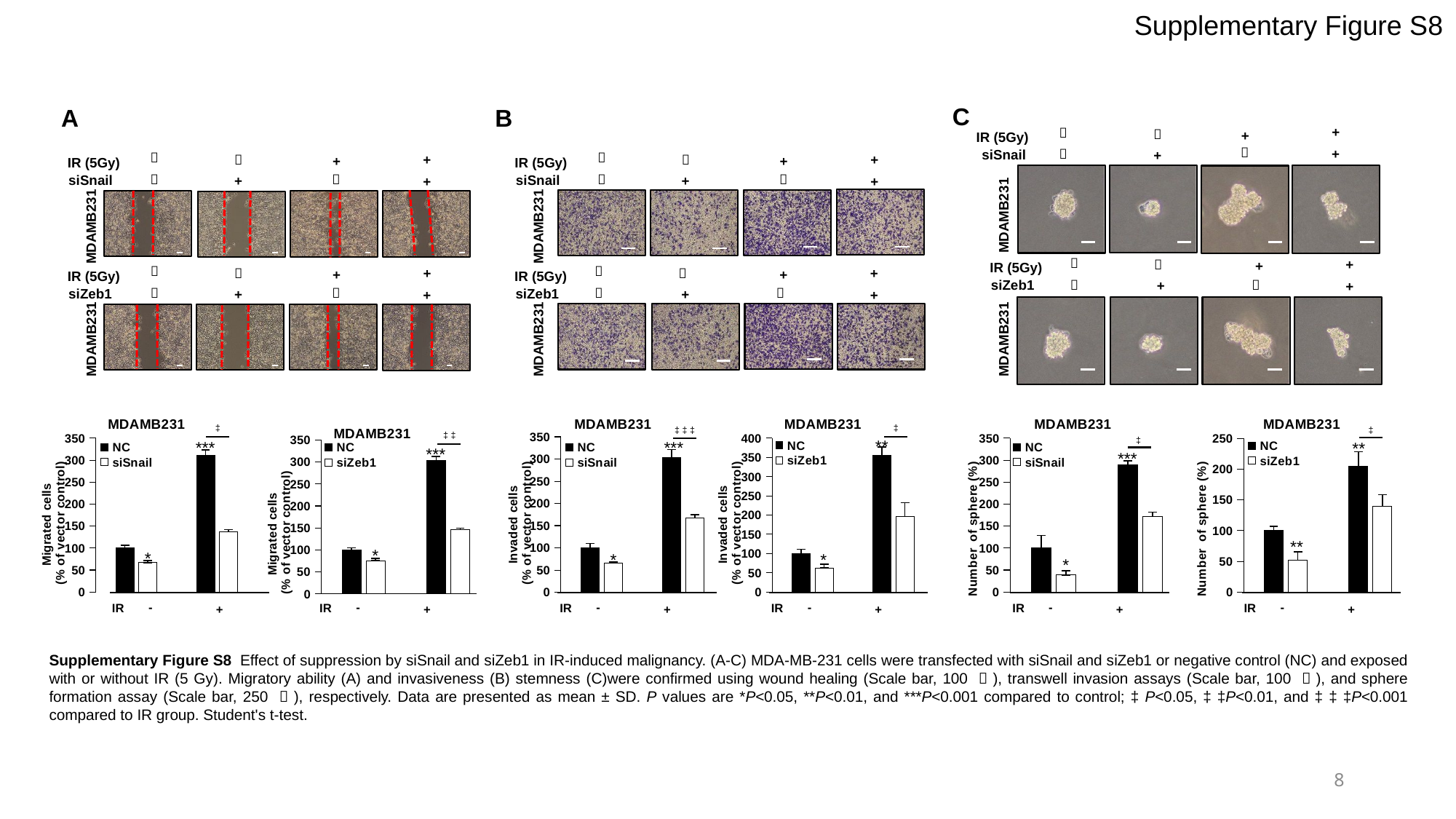

Supplementary Figure S8
C
B
A
+
ㅡ
ㅡ
+
IR (5Gy)
ㅡ
+
ㅡ
siSnail
+
MDAMB231
ㅡ
ㅡ
+
+
IR (5Gy)
ㅡ
ㅡ
siZeb1
+
+
MDAMB231
ㅡ
ㅡ
+
+
IR (5Gy)
ㅡ
ㅡ
siSnail
+
+
MDAMB231
ㅡ
ㅡ
+
+
IR (5Gy)
ㅡ
ㅡ
siZeb1
+
+
MDAMB231
ㅡ
ㅡ
+
+
IR (5Gy)
ㅡ
ㅡ
siSnail
+
+
MDAMB231
ㅡ
ㅡ
+
+
IR (5Gy)
ㅡ
ㅡ
siZeb1
+
+
MDAMB231
### Chart: MDAMB231
| Category | NC | siZeb1 |
|---|---|---|
| ㅡ | 100.0 | 75.70621468926552 |
| IR | 303.3898305084746 | 146.89265536723164 |‡ ‡
***
*
-
IR
+
### Chart: MDAMB231
| Category | NC | siSnail |
|---|---|---|
| ㅡ | 100.0 | 66.66666666666666 |
| IR | 310.4938271604938 | 137.03703703703704 |‡
***
*
-
IR
+
### Chart: MDAMB231
| Category | NC | siZeb1 |
|---|---|---|
| ㅡ | 100.0 | 62.430939226519335 |
| IR | 355.2486187845304 | 195.966850828729 |‡
**
*
-
IR
+
### Chart: MDAMB231
| Category | NC | siSnail |
|---|---|---|
| ㅡ | 100.0 | 38.88888888888889 |
| IR | 288.88888888888886 | 172.22222222222223 |‡
***
*
-
IR
+
### Chart: MDAMB231
| Category | NC | siSnail |
|---|---|---|
| ㅡ | 100.0 | 66.0377358490566 |
| IR | 303.3018867924528 | 166.698113207547 |‡ ‡ ‡
***
*
-
IR
+
### Chart: MDAMB231
| Category | NC | siZeb1 |
|---|---|---|
| ㅡ | 100.0 | 51.99999999999999 |
| IR | 204.0 | 140.0 |‡
**
**
-
IR
+
Supplementary Figure S8 Effect of suppression by siSnail and siZeb1 in IR-induced malignancy. (A-C) MDA-MB-231 cells were transfected with siSnail and siZeb1 or negative control (NC) and exposed with or without IR (5 Gy). Migratory ability (A) and invasiveness (B) stemness (C)were confirmed using wound healing (Scale bar, 100 ㎛), transwell invasion assays (Scale bar, 100 ㎛), and sphere formation assay (Scale bar, 250 ㎛), respectively. Data are presented as mean ± SD. P values are *P<0.05, **P<0.01, and ***P<0.001 compared to control; ‡ P<0.05, ‡ ‡P<0.01, and ‡ ‡ ‡P<0.001 compared to IR group. Student's t-test.
8
